# A Quantitative Systems Approach to Define Novel Effects of Tumour p53 Mutations on Binding Oncoprotein MDM2

**DOI:** 10.3390/ijms23010053

**Published:** 2021-12-21

**Authors:** Manuel Fuentes, Sanjeeva Srivastava, Angela M. Gronenborn, Joshua LaBaer

**Affiliations:** 1 Cancer Research Center (IBMCC/CSIC/USAL/IBSAL), Department of Medicine and General Cytometry Service-Nucleus, CIBERONC ISCIII, 37007 Salamanca, Spain; mfuentes@usal.es; 2 Cancer Research Center (IBMCC/CSIC/USAL/IBSAL), Proteomics Unit, 37007 Salamanca, Spain; 3 Department of Biosciences and Bioengineering, Indian Institute of Technology (IIT) Bombay, Mumbai 400076, India; sanjeeva@iitb.ac.in; 4 School of Medicine, University of Pittsburgh, 1050 BST3, Pittsburgh, PA 15260, USA; amg100@pitt.edu; 5 Virginia G. Piper Center for Personalized Diagnostics, Biodesign Institute, Arizona State University, Tempe, AZ 85287, USA

**Keywords:** p53-mdm2 interaction, protein microarrays, high-throughput label-free detection

## Abstract

Understanding transient protein interactions biochemically at the proteome scale remains a long-standing challenge. Current tools developed to study protein interactions in high-throughput measure stable protein complexes and provide binary readouts; they do not elucidate dynamic and weak protein interactions in a proteome. The majority of protein interactions are transient and cover a wide range of affinities. Nucleic acid programmable protein arrays (NAPPA) are self-assembling protein microarrays produced by freshly translating full-length proteins in situ on the array surface. Herein, we have coupled NAPPA to surface plasmon resonance imaging (SPRi) to produce a novel label-free platform that measures many protein interactions in real-time allowing the determination of the KDs and rate constants. The developed novel NAPPA-SPRi technique showed excellent ability to study protein-protein interactions of clinical mutants of p53 with its regulator MDM2. Furthermore, this method was employed to identify mutant p53 proteins insensitive to the drug nutlin-3, currently in clinical practice, which usually disrupts the p53-MDM2 interactions. Thus, significant differences in the interactions were observed for p53 mutants on the DNA binding domain (Arg-273-Cys, Arg-273-His, Arg-248-Glu, Arg-280-Lys), on the structural domain (His-179-Tyr, Cys-176-Phe), on hydrophobic moieties in the DNA binding domain (Arg-280-Thr, Pro-151-Ser, Cys-176-Phe) and hot spot mutants (Gly-245-Cys, Arg-273-Leu, Arg-248-Glu, Arg-248-Gly), which signifies the importance of point mutations on the MDM2 interaction and nutlin3 effect, even in molecular locations related to other protein activities.

## 1. Introduction

Systems biology and proteomics emerged as powerful tools for understanding protein interactions with different partners under various micro-environmental conditions. However, as this area is still evolving, generating a concrete understanding of the quantitative estimation of this dynamic behavior is still a daunting task. Such interactions often vary from stable-bonds, high to low-affinity complexes, and short-lived interchanges under different environmental conditions. The majority of protein-protein interactions are transient in nature involving an unstable intermediate complex which is difficult to capture using conventional methods [1,2,3]. Therefore, there is a pressing need to develop more advanced tools in order to measure such transient interactions at higher resolutions necessary to elucidate the mechanistic conundrum of the dynamic proteome related to its affinity with different partners and kinetics of those interactions. 

The majority of the conventional methods like co-immunoprecipitation (Co-IP), two-hybrid approaches, and tandem affinity purification (TAP) prior to mass spectrometry are more suitable to analyze the stable protein complexes; dynamic weak interactions do not survive long enough for detection. These techniques are employed in understanding moderate to HT studies, though the extrapolation of those data in order to resolve dynamic interaction that can be manipulated by extrinsic forces remains still a challenging area [1,2,3,4]. Moreover, these techniques can only provide coarse information about binding affinity and do not record the kinetics behavior of transient interactions. 

Protein microarrays are an attractive emerging technology for understanding protein interactions. Interestingly, protein microarrays allow the simultaneous study of hundreds to thousands of proteins displayed in high spatial density in a microscopic format [5]. As an open biochemical platform, they can be manipulated to obtain real time binding data and thus amenable to study of binding kinetics. A challenge for protein microarrays is ensuring availability of well-folded and relevant protein content. 

The traditional manufacture of protein microarrays entails printing purified proteins onto a substrate, which requires the purification, storage, and manipulation of thousands of proteins. This is a time-consuming, tedious, costly process and often results in uneven display protein yields that span more than 3 orders of magnitude, most of which is low. Moreover, the manipulation and storage of the proteins may sometimes lead to loss of function. In our previous reports, we have addressed the yield and activity-related issues by employing a more advanced Nucleic Acid Programmable Protein Arrays (NAPPA) technology. Unlike conventional microarray, in NAPPA the process of printing of proteins is replaced with the simple process of spotting cDNAs that encode the proteins fused to a common binding tag. At the time of the experiment, these cDNAs are simultaneously transcribed/translated in situ with mammalian ribosomes and the expressed proteins are subsequently captured using the c-terminal epitope tag. Importantly, this approach eliminates the need for HT protein isolation, while allowing to achieve highly consistent levels of all protein types and ensures that all the proteins are produced in full-length and freshly synthesized [6,7]. 

Typically, the interactions on protein microarrays have been detected with fluorescently-labeled antibodies or tagged ligands after multiple buffer washes, thus recording steady-state binding only. However, most variants of protein microarrays are also compatible with label-free approaches, which are capable of measuring dynamic protein interactions. The benefit of coupling protein microarrays with a label-free, real-time technology is a tool with the immense power of measuring the dynamics of protein interactions against numerous proteins simultaneously.

Surface plasmon resonance (SPR) has evolved as one of the most appealing methods for measuring various dynamic molecular interactions by detecting even minute changes in the localized refractive index induced by the binding of an analyte (test protein) molecule to an immobilized target (such as metallic sensor surface). Changes in the SPR signal correspond to the dynamic interaction of the test protein with the immobilized target thus allowing the capture of real-time assessment of dynamic protein interactions [8,9,10]. Most commercially available SPR sensors utilize a focused set of independent sensing channels. These require that purified target protein be immobilized in the channel using a complex process that must avoid denaturation, maintain function and achieve high reproducibility. The complexity of the setup and the requirement for purified protein frequently requires the use of peptides instead of full-length proteins. These systems are useful for performing detailed studies on a small set of analytes but are not practical for HT applications.

A recent adaptation of standard SPR technology resulted in an SPR-based imaging (SPRi) tool that showed huge potential for the simultaneous measurement of thousands of biomolecular interactions [9,10,11]. Instead of scanning the surface at different angles, the SPRi measurements are performed at a fixed angle of incidence set in the linear region of the SPR dip, such that changes in reflected light intensity induced by the binding of biomolecules are proportional to the changes in refractive index. The naïve binding, steady state and washing off of ligand can be captured and analyzed from a movie of the data. Owing to the fact that SPR is a label-free technology, the query binding can be monitored without making use of fusion tags or antibodies. This approach not only significantly minimizes the efforts and cost but simultaneously avoids various artifacts that can potentially be introduced through such tags [8,9,10,11].

Herein, we report the development of a novel protein microarray-driven SPRi platform which is based on our previously developed NAPPA technology. Newly developed NAPPA-SPRi technology is specifically designed to make a facile comparison of the kinetic information on both the strong as well as weak interactions without the need of having to purify each target protein. Importantly, our method is capable of measuring those interactions at a scale that was not attainable earlier with previously developed techniques. In the current report, we have demonstrated our method in elucidating a mechanistic understanding of the dynamic interaction of p53 protein with MDM2 in the presence and absence of nutlin-3. Nutlin-3 is a potent antitumoral drug already in the clinic. Intracellular action of nutlin-3 as an inhibitor of p53-MDM2 binding is well characterized in the literature. Additionally, we have also described protein-protein interactions of known clinical mutants of p53 with its regulator MDM2. The TP53 gene is the most commonly mutated gene in cancer, as it is mutated in greater than 50% of all cancers. Greater than 95% of these mutations are missense mutations that alter the function and regulation of p53 [4]. Mutations in TP53 increase the stability of p53 protein, leading to an accumulation and increase in antigen presentation of this highly immunogenic protein. Interestingly, our study revealed that the mutant p53 proteins remain largely insensitive to the drug nutlin-3, which usually disrupts the p53-MDM2 interaction.

## 2. Results and Discussion

### 2.1. The NAPPA-SPRi Array

**Figure 1:**
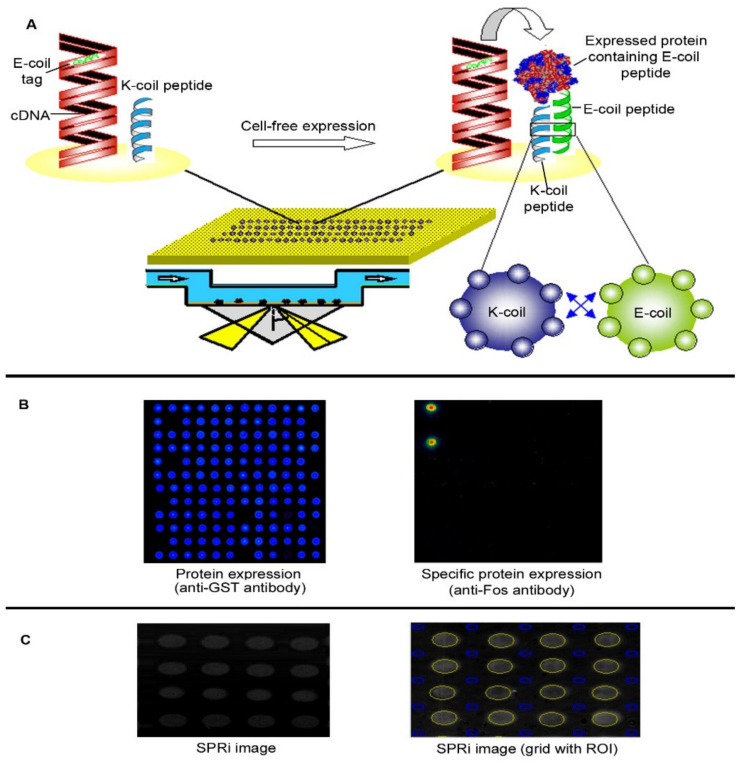
Integration of NAPPA with SPRi. (**A**) Full length cDNAs, including E-coil tags encoded at their C-termini, and K-coil peptide are co-printed on gold surfaced microscope slides bearing an amino-PEG self-assembled monolayer. At the time of experimentation, the corresponding proteins were expressed using rabbit reticulocyte lysate-based cell-free expression system. The in situ synthesized proteins were captured by the surface bound K-coil peptide, which specifically dimerizes with the E-coil tag based on a coiled-coil mechanism. (**B**) This test array, which included duplicate features of wild type and 46 clinical point mutants of the tumor suppressor p53, was confirmed for protein expression and capture using fluorescent detection of a universal anti-tag antibody (anti-GST antibody) and an anti-Fos antibody, respectively. The negative control for protein expression included a non-expressing plasmid. (**C**) Working in the linear region of the SPR curve enabled the quantitative correlation between changes in reflected light intensity with the amount of material on the surface. Left. Raw CCD image of the microarray features displaying protein in running buffer. Oval feature shape resulted from the shallow camera angle. Right. SPRi image of the array with software-marked regions of interest (ROI) (yellow circles). Each ROI is surrounded by 4 background reference spots (blue circles).

Surface plasmons require metallic surfaces, such as gold. We initially adapted our standard NAPPA chemistry for gold-coated microscope slides by functionalizing the gold with amino-PEG-thiols which resulted in the formation of a homogeneous self-assembled monolayer of gold on microscopic slides (see Supplementary Figure S1). This approach allowed seamless fluorescent detection; however, the accumulated mass of bovine serum albumin (BSA), antibodies, and DNA that are used in our NAPPA chemistry limited measurement of mass changes from ligand binding, which were small compared to the already-bound mass. In order to reduce the starting mass, we developed a novel capture chemistry approach by replacing the antibody-based method with a pair of peptides that interact via a dimerization mechanism using synthetically designed coiled-coils (Figure 1), referred to as E-coil and K-coil, that heterodimerize with 30 pM affinity [12,13]. The E-coil sequence was inserted as a C-terminal fusion tag along with the GST protein, which also helped to space the protein away from surface (see Supplementary Figure S2). Purified K-coil peptide was printed on the array surface along with the cDNA. Protein expression and capture were verified using anti-GST antibodies and standard fluorescent detection. The ability to detect protein at the features by SPRi was confirmed by examining the SPR image (Figure 1).

**Figure 2:**
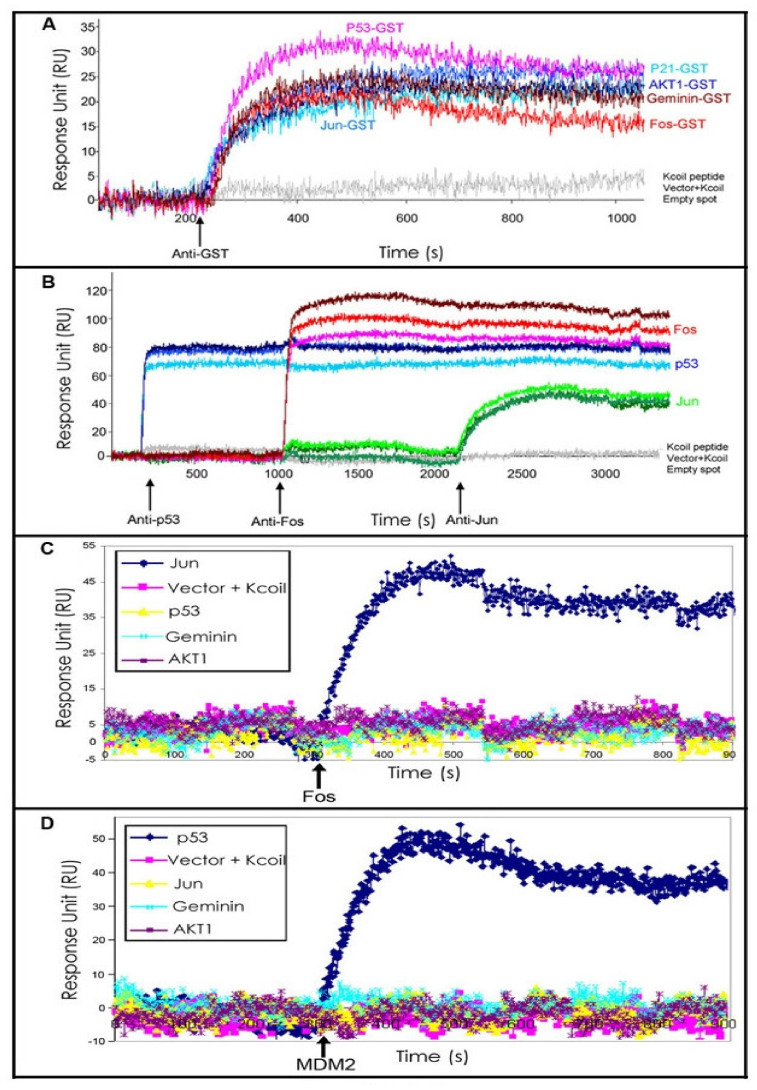
Real-time label-free detection of protein interactions in high-throughput format by NAPPA-SPRi. (**A**) The indicated proteins with C-terminal GST E-coil tags were expressed according to the NAPPA method, placed on the SPRi instrument and CCD recording initiated. The proteins were probed with the anti-GST monoclonal antibody at the time indicated and the individual sensorgrams were identified based on their known location on the array. Sensorgrams corresponding to empty spots, plasmid vector alone and the capture peptide K-coil did not exhibit any binding event. (**B**) Specific protein detection was illustrated by probing a NAPPA-expressed array bearing GST-E-coil tagged proteins printed in triplicate with protein-specific antibodies. Monoclonal anti-p53, anti-Fos, and anti-Jun antibodies were injected sequentially as indicated and revealed binding only to their relevant targets. (**C**) The sensorgrams for the indicated protein features that were observed when purified recombinant Fos protein were used as query to probe a NAPPA array that included its known binding partner Jun followed by a wash with running buffer at 550 s. (**D**) An experiment similar to that in (**C**) using purified recombinant protein MDM2 as query and detecting the p53-MDM2 binding event using SPRi. The blue line indicates the time when the running buffer was added.

To test whether binding to NAPPA expressed proteins could be detected by SPRi, an array was printed with plasmids coding for the human proteins geminin, Akt1, fos, p53, and jun along with the GST-E-coil tag at their C-termini and expressed in situ. Figure 2A shows the sensorgram after the anti-GST antibody (20 μg/mL) was injected into the flow cell. Anti-GST antibody showed excellent binding to all the constructs encoding proteins with the GST moiety, but none of the negative controls included K-coil peptide, empty vector, or water. A duplicate array showed the same pattern of detection by standard fluorescence methods (data not shown). To demonstrate specific protein detection, an array in which these proteins were all expressed in triplicate was sequentially injected with specific monoclonal antibodies specific to p53, fos, and jun (Figure 2B). The sensorgram demonstrated specific binding on the features that corresponded to each specific protein with a little non-specific binding or cross-talk (Supplementary Figure S3).

Similarly, we further tested this configuration for non-antibody-based protein-protein interactions by examining the binding of the transcription factor fos (query) to its partner jun (target on the array; Figure 2C) and the binding of the full-length E3 ubiquitin ligase MDM2 (query) to p53 (target; Figure 2D). Fittings of the sensorgrams for independent experiments were highly reproducible (Supplementary Figure S4). 

Our computed KD for the fos-jun interaction was 0.29 ± 0.01 nM (Supplementary Figure S5). Although there are no reported values for the KD of this interaction using an SPR platform, other methods, including both co-IP and isothermal calorimetry on peptides and protein fragments, have reported the affinity to be 1.0 ± 0.3 nM [14].

MDM2 forms a dimer [10,11,12]; therefore, using full-length protein as a query in SPRi yields an “apparent affinity constant” that represents both binding site affinity and avidity, hereafter referred to as the KD. The apparent affinity constant of the p53-MDM2 interaction using full-length proteins obtained in our approach was 85 ± 2 nM (Supplementary Table S1). Although there are no published values for the affinity of the MDM2-p53 interaction for full-length proteins, this binding was in a similar affinity range as was estimated by different techniques using p53 peptides and MDM2 fragments, 60 nM [15,16,17,18]. 

### 2.2. p53 Mutant—Mdm2 Interaction Studies 

The p53 tumor suppressor protein comprises a trans-activation and proline-rich domain, a DNA-binding domain, and a tetramerization and regulatory region. The p53 forms a tetrameric transcription factor that regulates central biological functions [19,20,21] (Supplementary Figure S6). Mutations in p53 gene are one of the most frequently observed genetic alterations in human cancer and have been identified in more than 100 different codons, implying that diverse structural changes in p53 can promote carcinogenesis [20,21]. In unstressed conditions, p53 abundance is post-transcriptionally regulated by MDM2, which ubiquitylates p53 for constitutive degradation by the proteFigureasome [15,16]. The disruption of the p53-MDM2 auto-regulatory feedback loop is a current therapeutic target intended to elevate p53 levels. Nutlin-3, a cis-imidazoline analog, displaces recombinant p53 from the p53-MDM2 complex with an IC50 in the 100–300 nM range [22]. In vitro and in vivo data suggest that nutlins could be potentially developed for use as anti-tumor drugs [19,22,23]. However, some cell lines containing mutant forms of p53 are resistant to or only weakly affected by nutlins (Supplementary Table S3). We, therefore, assessed the interactions between clinically relevant p53 mutations, MDM2 and nutlin-3. These mutations primarily map to the DNA binding domain of p53 and thus presumably affect its role as a transcription factor, although additional functional changes may also occur.

We assembled a NAPPA array containing constructs encoding 46 of the most common p53 point mutations. This p53 protein array displayed each mutant in duplicate and in different zones of the array. In addition, the array contained 36 duplicate control features including K-coil peptide, empty expression vector, water, unmodified surface, and features displaying the fos protein. Protein display was confirmed for all proteins using anti-GST antibody with fluorescent detection (Figure 1B).

**Figure 3:**
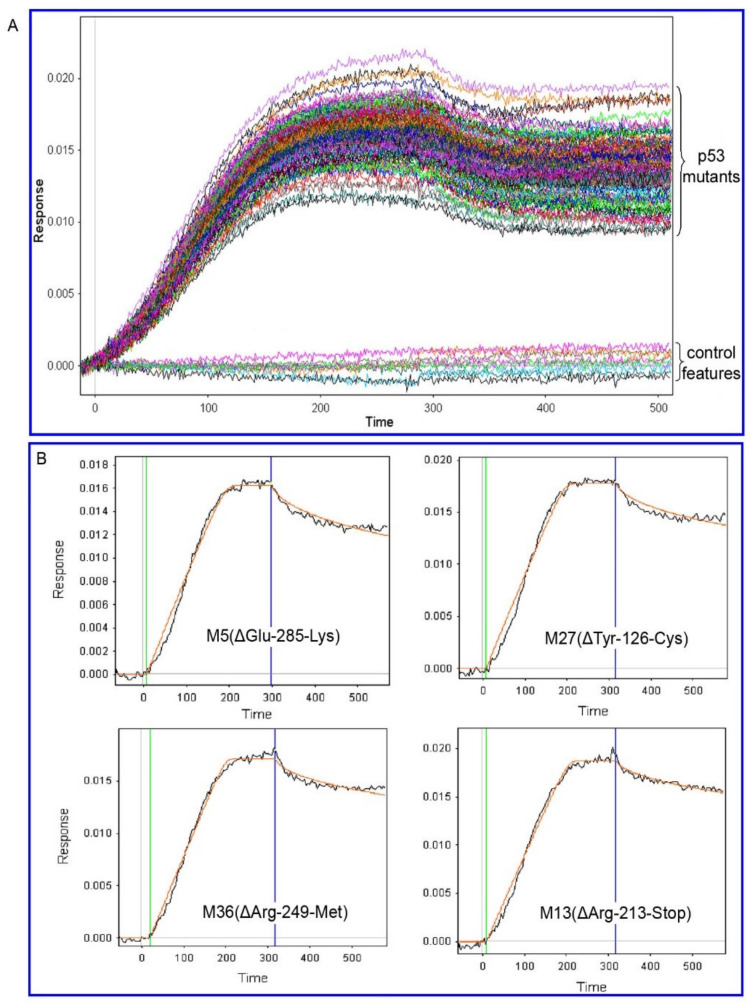
Real-time label-free detection of protein interactions in high-throughput format by NAPPA-SPRi. Simultaneous real-time detection of the interaction between Mdm2 and various p53 species in a high-throughput format by NAPPA-SPRi. (**A**) Sensorgrams for a NAPPA array containing duplicate features of wild type p53 and 46 common clinical mutants that were prepared and placed on the SPRi device. Full-length purified recombinant MDM2 in running buffer was added and then followed by running buffer alone at 250 s. Binding was observed for p53-specific spots but none of the control features. (**B**) Examples of individual sensorgrams from (**A**) that were fitted to a two-component model (association and dissociation) by Scrubber 2.0 to produce kinetic parameters. The orange line corresponds to the fit curve, the green line corresponds to the injection point starting the protein association, and the blue line corresponds to the dissociation stage of the protein interaction after the stochastic steady state. Note that the arrival of binding query protein at one end of the array before the other end results in slightly different binding start times from one protein feature to the others.

We evaluated the real-time interaction of these p53 mutants on the array with full-length, wild-type MDM2 using SPRi, as demonstrated in the sensorgram (Figure 3). We observed that binding was specific for p53-spots with no binding on control features. The fitted curves for several representative sensorgrams are shown in Supplementary Figure S7. To simplify the analysis, and because we did not evaluate the mutants that disrupt tetramerization, these sensorgrams were fitted in a 1:1 kinetic mechanism model using Scrubber 2.0 with a minimum residual of ≤0.001, which enabled scaling of values for the mutants [24]. From these data, we were able to calculate the kon, koff, and KD for most of the p53 proteins tested, although some values fell outside of the range of the instrument. The KD values that were calculated ranged from 2.85 nM to 1.35 µM with a median value of 105 nM (Supplementary Table S1). The results showed a wide range of KD values from nM to the low µM range. Like wild-type protein, most of the mutants showed affinities the single to double-digit nanomolar range, whereas only a small fraction had significantly weaker binding. 

### 2.3. p53-Mdm2 Interaction Studies in the Presence of Nutlin-3

**Figure 4:**
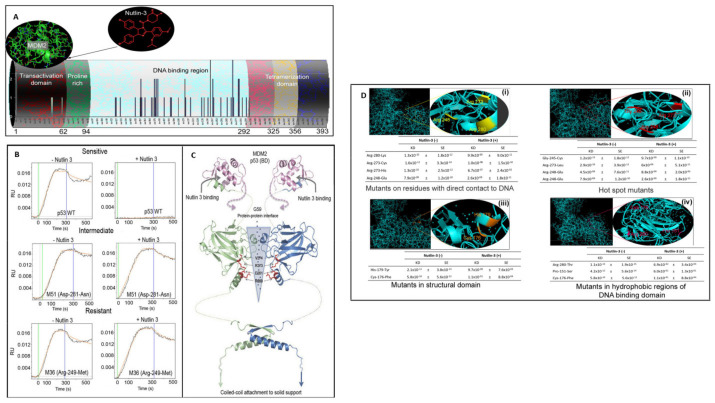
NAPPA-SPRi to monitor the effect of nutlin-3 on the binding of MDM2 to mutant and wild-type p53. (**A**) Schematic representation of the p53-MDM2-nultin-3 model. (**B**) An experiment was performed as described in Figure 3, but in the presence of nutlin-3 (900 nM), an inhibitor of the MDM2-p53 interaction. The sensorgrams indicate the binding of MDM2 to p53 in the presence and absence of nutlin-3 as indicated. Examples of individual sensorgrams of sensitive, intermediate and resistant p53 features that were fitted to a two-component model are demonstrated. (**C**,**D**) KD value for p53-MDM2-nultin3 interactions at different mutant locations on p53 protein structure (i–iv).

As some mutations in p53 clearly affected its interaction with MDM2, and considering that cell lines that reported certain p53 mutations fail to respond to nutlin-3, we investigated the effects of the mutations on the ability of nutlin-3 to disrupt the p53-MDM2 interaction. As expected, the binding of MDM2 to wild-type p53 was profoundly affected by the presence of nutlin-3 (900 nM; Figure 4), reducing the binding affinity by ~5 orders of magnitude (Supplementary Table S2). This data is well-correlated with functional studies of numerous cell lines that constitutively express p53, which undergo cell-cycle arrest after nutlin-3 prevents the proteolysis and thereby activates p53 (Supplementary Table S3) [25,26,27,28,29,30,31,32,33,34,35,36,37,38,39,40,41,42,43,44,45,46,47,48,49,50,51,52,53,54,55,56].

On the same protein microarray, we simultaneously assessed the effects of the various p53 mutations on the ability of nutlin-3 to disrupt p53-MDM2 binding. By examining the ratio of the KD in the absence and presence of nutlin-3, we observed 3 classes of disruption response: strong, intermediate, and no effect (Figure 4A,B and Supplementary Table S3). The majority of mutants fell into the strong group, which like wild type p53, revealed a profound average change in KD of 5.7 orders of magnitude with nutlin-3. There was a good correlation between mutants that were disrupted strongly by nutlin-3 in vitro and cell lines harboring those mutations that were sensitive to nutlin-3-mediated cell cycle arrest. Table 1 includes a subset of the mutants tested here for which cellular response data were available in the literature (see also Supplementary Table S3) [26,27,28,29,30,31,32,33,34,35,36,37,38,39,40,41,42,43,44,45,46,47,48,49,50,51,52,53,54,55,56]. These highly correlated values, while not a direct validation, show consistency between the SPRi platform results and reported biological observations.

A number of the mutants showed no disruption by nutlin-3 of the p53-MDM2 interaction, including no significant effect on kon, koff, or KD (Figure 4). As indicated in Table 1, cell lines harboring these p53 mutations have been reported to be resistant to the effect of nutlin-3 (30 µM) for cell cycle arrest [26,27,28,29,30,31,32,33,34,35,36,37,38,39,40,41,42,43,44,45,46,47,48,49,50,51,52,53,54,55,56].

Interestingly, we observed a few intermediate mutants with weak effects of nutlin-3 on the protein interaction, which reduced the KD on average by 2.3 orders of magnitude. We could not discern a specific pattern regarding the contribution of kon or koff to this nutlin-3-induced loss of binding affinity, only that the net effect on KD was not as profound as in the strong class and there was no marked effect on koff. Cell lines that express these specific mutations have shown a cell cycle response to nutlin-3 but only at higher concentrations (Table 1 and Supplementary Table S3) [26,27,28,29,30,31,32,33,34,35,36,37,38,39,40,41,42,43,44,45,46,47,48,49,50,51,52,53,54,55,56]. 

As all of these measurements were done side-by-side under the same conditions, it is useful to compare them from one to the other. No simple pattern explained how all of the mutations affected the binding constant, which is naturally the case in mutant screening experiments as mutations can affect either local binding or global structure. Here, the focus on common clinical mutations meant that none of the mutations tested are located in the MDM2 binding helix of p53 (residues 19–26), thus effects in the direct binding pocket are not considered. 

It is useful to compare the effects of point mutations on the p53-MDM2 interaction first in the absence and then in the presence of nutlin-3 (Supplementary Table S1). If no substantive difference is observed between the wild type and mutant interaction with MDM2, such as seen for the Arg249Met or His179Arg and His179Tyr mutants, but a substantial nutlin-3 effect is observed, as is the case for His179Tyr, it is most likely that nutlin-3 allosterically interferes with MDM2 binding. If, on the other hand, no nutlin-3 effect or a marginal nutlin-3 effect is observed as for His179Arg, this protein complex is not conformationally affected by nutlin-3. The pair His179Arg/His179Tyr is an illustrative example for an allosteric effect—in both mutants His179 is changed (for Arg and Tyr, respectively) and from the interaction data with MDM2 in the absence of nutlin-3, no substantial difference was noted (thus the overall structure is essentially intact); however, clearly nutlin-3 has no effect on His179Arg, suggesting that the nutlin-3 effect on the His179Tyr mutant is an allosteric effect. In structural terms, a possible explanation may involve the nature of the side-chain substitution: a His-Tyr change is a conservative change (aromatic to aromatic) while the His to Arg change is more dramatic. Thus, if an aromatic interaction is involved in the allosteric effect caused by nutlin-3, the Tyr mutant would be able to substitute better than the Arg mutant. 

From all available structural data [20,57,58,59,60,61], it has become clear that very similar interaction surfaces are used on the p53 core domain when binding to DNA and other proteins. Thus, this binding interface (schematically illustrated in Figure 4C, with several side chains of mutants highlighted) naturally would be considered when evaluating possible allosteric effects. p53 binding to MDM2 can be modulated by mutation, possibly in an allosteric-like way. It has been shown previously that the binding affinity of MDM2 can be allosterically modulated through the RING finger domain and the increment in affinity goes hand-in-hand with MDM2 transrepressor activity [62]. This allosteric model was supported by differences in protein conformation and pocket accessibility in wild-type and the RING domain mutant MDM2 proteins [62]. Similarly, it was found that the small molecule inhibitor RITA binds outside of the MDM2 binding site to p53, suggesting that p53/MDM2 interaction is abrogated by RITA via an allosteric mechanism [26,27,28,29].

Interestingly, several nutlin-3 sensitive mutations reside in this interface, and the effects of nutlin-3 on the mutant Arg280Thr as well as the mutant Asp281Asn could reflect structural changes in this protein-protein interface, with Arg280Thr exhibiting a strong effect compared to an intermediate effect on Asp281Asn. In this case, MDM2 binding to Asp281Asn is somewhat perturbed (reduced), possibly indicating an overall destabilization of the mutant protein. Among the nutlin-3 resistant mutants, Arg273Leu represents an interesting case: the interaction with MDM2 is unperturbed (essentially similar binding parameters as p53wt; Supplementary Table S1). Therefore, this particular mutant appears to have an altered side chain that is not important in the putative allosteric interaction involving a protein-protein interface. It is well established that the Arg273 side-chain provides one of the most pivotal interactions on the p53 core domain with DNA [20]. Thus, if as hypothesized here, the nutlin-3 effect is mediated via protein-protein interaction, the nutlin-3 resistance of this mutant could be explained in a straightforward manner. Naturally, not all mutants yield such clear results and further dissection of the MDM2 binding data in conjunction with the nutlin’s effect is necessary. 

Here, we describe a HT method to follow the real-time interaction of a query protein with many full-length target proteins, which avoids the need to produce and purify the target proteins. A particular advantage is the ability to study full-length proteins where this has been challenging to achieve by conventional techniques. It enables the measurement of binding kinetic data for many proteins simultaneously without the need for labels or specific antibodies to the query protein. Moreover, the ability to produce in situ proteins avoids multiple protein purifications followed by the tedious attachment of the purified proteins to the array. The measurement of the KD was highly reproducible over replicate measurements. The median standard error (SE) was <0.3% of the KD value. There was more variability in the rate measurements, with median SEs ranging from 0.3–25% of the measured values. This method is ideal for determining relative quantitative values amongst numerous proteins. Refinement of these values to achieve precise and accurate absolute values currently still requires more detailed studies in lower throughput. Yet, there are numerous applications that would benefit from an early and rapid assessment of relative binding affinities amongst many candidates. 

This represents a facile method for the HT study of transient interactions. Applications include assessing a query’s interaction with a group of proteins of interest, such as members of a particular pathway or family, or it can be applied in a more detailed study of various relevant mutant or modified forms of a single protein, as demonstrated here. It will be useful to apply this approach to characterize the structure-activity relationships of proteins, providing an adjunct tool in the quest for more personalized approaches. These kinetic data will enhance our understanding of protein function at an accelerated pace. However, improved informatics methods and tools will be needed to manage the analysis and interpretation of the voluminous data generated by this approach.

## 3. Materials and Methods

### 3.1. DNA Preparation and Substrate Functionalization

Bacteria harboring the p53 expression plasmids (pANT7-GST-Ecoil vector, clon ID EvNO00023103 (DNASU Plasmid Repository www.dnasu.org (accessed on 10 October 2012)), (Supplementary Figure S2) were cultured in 150 mL of Luria-Bertani medium containing 10% potassium phosphate and 100 μg/mL ampicillin in 96 well-plates for 24 h, then pelleted by centrifugation at 2500× *g* for 20 min. DNA was prepared according to Qiagen MaxiPrep Kit and concentration was measured by spectrophotometer followed by agarose. 

Nanocapture gold activated slides (25 × 76 × 1 mm slide covered with a thin layer of gold 47.5 nm) were obtained from Plexera (USA). The gold surface was functionalized by a 1 mM solution of amino-PEG-thiols (HS-(CH2)11-EG6-NH2 supplied by Prochimia (Poland) to generate a homogeneous self-assembled monolayer onto the gold surface. The SAM preparation was performed in a tight box filled with ethanol. Gold slides were placed on a metal rack and after amino-PEG-thiol treatment, the coverslip was placed. The humidifier chamber was closed and after 16 h of incubation, the slides were washed with ethanol and dried using filtered air (see Supplementary Figure S1). Gold-SPR slides were then stored in a dry container with silica packs until ready for printing. 

### 3.2. Sample Preparation and Array Printing

DNA was prepared and quantified as described previously. Briefly, 90 μg of DNA sample was precipitated by addition of 0.8× volume of isopropanol and centrifugation at 4000× *g* for 30 min. Precipitated DNA was then washed with 80% ethanol and allowed to air dry. K-coil peptide (sequence: GGGnLKSALKEKVSALKEKVSALKEKVSALKEKVSALKE-N-terminal acetylated, C-terminal amide, nL = Norleucene) was purchased from New England Peptides (Gardner, MA, USA) (Supplementary Figure S2). The master mix solution was made up by mixing 300 μM of K-coil dissolved in water and 90 μg of cDNA encoding each of the p53 mutants. The master mix was transferred to a 384 well microtiter plate. 

The master mix plate and the SF10 gold slides (Plexera Inc., Seattle, WA, USA) were loaded to a Qarray2 robotic microarray spotter (Genetix Inc., San Jose, CA, USA) configured to use 48 pins that produced 300 μm features. Microarrays were printed by pin-spotting master mix solution on NanoCapture Gold SPR-functionalized slides. The relative humidity was maintained at 60% during the printing. The printing program was set to print arrays in 12 rows × 12 columns and each feature was printed in duplicate. The master mix was also printed on gold slides (Gentle Inc., Madison, WI, USA) and amino silane (Pierce Co., Rockford, IL, USA) functionalized glass slides. After printing was completed, the arrays were stored in a dry container with silica packets. 

### 3.3. In Situ Protein Expression in NAPPA-SPRi Approach

The printed gold arrays were washed with 1× PBS for 15 min with gentle agitation, followed by a brief washing step with deionized water for 1 min. The array surface was blocked with a solution of sulfate-dextran (Sigma Inc., St. Louise, MO, USA) for 1 h at RT with gentle agitation and followed by 5 min wash with deionized water. The arrays were dried under a stream of filtered compressed air. 

The in vitro transcription and translation step was performed as previously described [6]. Briefly, the HybridWell (Grace Biolabs Inc., Bend, OR, USA) gasket was applied to the slides. The in vitro transcription-translation lysate mix was prepared with 200 μL of reticulocyte lysate (Promega, Madison, WI) containing 16 μL of TNT buffer, 8 μL of T7 polymerase, 4 μL of –Met, 4 μL of –Leu or –Cys, 8 μL of RNaseOut (Invitrogen Inc., San Jose, CA, USA), 160 μL of DEPC water. The mix was added onto the slide and HybriWell gently massaged to spread out the mix uniformly on the array. Port seals were applied to both ports on HybriWell to avoid evaporation. The arrays were incubated for 1.5 h at 30 °C and 30 min at 15 °C for protein expression and captured by immobilized K-coil, respectively. The HybriWell was removed, and the array was washed with 1× PBS for 5 min on a rocking platform.

### 3.4. Protein Display and Visualization

Prior to the SPRi experiments, we performed a few QC experiments to ensure the printing quality as well as protein expression on arrays. The printed arrays were blocked with superblock for 1 h on the rocking shaker. The PicoGreen (Invitrogen Inc., San Jose, CA, USA) staining was performed by applying 150 μL of 1:600 diluted PicoGreen (Figure 1). 

To detect protein expression universally on the array, the slides were incubated with superblock for 1 h. The mouse anti-GST antibody with a final solution of 10 μg/mL (Cell Signalling Technologies Inc., Danvers, MA USA) was applied on slides and incubated for 1 h at RT and a coverslip was placed. For detection of specific proteins, the slides were incubated with anti-p53 (DO-1) monoclonal antibody, anti-c-fos, and anti-jun antibodies (Santa Cruz Inc., Santa Cruz, CA, USA). After 1 h of incubation with primary antibody the slides were rinsed with superblock for 5 min × 3. The HRP linked secondary antibody with a final concentration of 10 μg/mL (anti-mouse IgG, Amersham Inc. Buckinghamsire, UK) was applied on slides and incubated for 1 h at RT and a coverslip was placed. After 1 h of incubation, the slides were rinsed with 1× PBS for 5 min in triplicate. 

The signal on arrays was developed by adding 600 μL of the tyramide signal amplification reagent (Perkin Elmer Inc., Waltham, MA, USA) for 10 min and a coverslip (Lifter slips Erie, Thermo Inc., Rockland, IL, USA) was applied. The slides were rinsed with de-ionized water, dried using compressed air, and scanned with a ProScanArray HT scanner (Perkin Elmer Inc., Waltham, MA, USA). The array images were quantified using Micro Vigene software version 2.9.9.2 (VigeneTech Inc., Carlisle, MA, USA). 

### 3.5. SPRi Instrument Set-Up

The SPRi analysis was performed on a ProteomicProcessor Instrument (Plexera, Seattle, WA, USA). The 1× PBS and 1% Tween 20 was degassed (50-kilopascal vacuum, 30 min) and used as SPR running buffer. 

The rate of data acquisition was set to 10 Hz. An elliptical region of interest (10 pixels height × 16 pixels width) was defined for each spot. Six background regions of interest of 10 × 16 pixels were defined at the top, middle, and bottom areas of the array. A simple two-point calibration was obtained by injecting 1× PBS (*n* = 1.3347 RIU) and 2× PBS (*n* = 1.4465 RIU) into the flow cell and recording the signal intensity at each point. The typical calibration factor was 2 camera units/micro refractive index unit (μRIU). Using a titration of glycerol solutions, the sensitivity of the instrument was determined as described by Campbell et al. [4]. The detection limit 0.3 ng/cm^2^ and <50 fg/spot, was determined. In this set of experiments, the Region of Interest (ROIs) was defined by SPRit software as per the manufacturer’s instructions (Plexera, Seattle, WI, USA).

### 3.6. Detection of In Situ Expressed Protein by SPRi

All the in situ expressed proteins contain GST-E-coil tag. The expressed protein was captured on the array due to the heterodimer interaction of E-coil with K-coil. The SPRi instrument was set up as previously described. The flow chamber was assembled onto the blocked slides and 5 mL of running buffer was injected with a flow rate of 10 μL/s. Once the baseline was stabilized, the minimal angle was set up. In this set of experiments, the minimal angle was approximately 20 ± 1 mm. At this angle, the ROIs were adjusted again in order to simultaneously measure the resonance units change of 144 spots. 

The detection of in situ expressed proteins was performed by anti-GST antibody (mouse anti-GST, Santa Cruzinc. Santa Cruz, CA, USA). After 250 s, 500 μL of anti-GST antibody solution (1:50 dilution in running buffer) was injected at a constant flow rate of 3 μL/s. At the end of the injection, a running buffer was applied for 1000 s at 10 μL/s. 

The specific detection of in situ expressed proteins (p53, jun, fos) was performed by anti-p53 (mouse anti-p53 DO-1, Santa Cruz Inc., Santa Cruz, CA, USA), anti-jun (mouse anti-jun, Santa Cruz Inc., Santa Cruz, CA, USA), anti-fos (mouse anti-fos, Santa Cruz Inc., Santa Cruz, CA, USA) antibodies. After 250 s, 500 μL of each antibody solution (1:50 dilution in running buffer) was injected at a constant flow rate of 3 μL/s. At the end of the injection, a running buffer was applied for 1000 s at 10 μL/s. 

### 3.7. Protein Interaction Studies by SPRi

To test p53-MDM2 and Jun-Fos interaction studies using SPRi, in situ protein expression and instrument set-up were performed as described above. After which, the flow chamber was assembled onto the gold chips and 5 mL of running buffer was injected with a flow rate of 10 μL/s. Once the baseline was stabilized, the minimal angle was set up. In this set of experiments, the minimal angle was approximately 20 ± 1 mm. At this angle, the ROIs were adjusted again in order to simultaneously measure the resonance units change of all the 144 spots printed on the array. 

The purified proteins Fos (Santa Cruz Inc., Santa Cruz, CA, USA) and Mdm2 (Calbiochem Inc., San Diego, CA, USA) were diluted in running buffer at a final concentration of 20 μg/mL. This solution was injected at a constant flow of 3 μL/s, and at the end of the injection, a running buffer was applied for 1000 s at flow rate of 10 μL/s. 

### 3.8. Protein Interaction Studies in the Presence of Nutlin-3 Using SPRi

To test the effect of nutlin-3 on p53-MDM2 interaction using SPRi, in situ protein expression and SPRi instrument set-up was performed as described above. After which, the flow chamber was assembled onto the gold chips and 5 mL of running buffer was injected with a flow rate of 10 μL/s. Once the baseline was stabilized, the minimal angle was set up. In this set of experiments, the minimal angle was approximately 20 ± 1 mm. At this angle, the ROIs were adjusted again in order to simultaneously measure the resonance units change of 144 spots. 

At a constant flow (3 μL/s) a solution of pure Mdm2 (20 μg/mL, Calbiochem Inc., San Diego, CA, USA) and 900 nM of nutlin-3 (Calbiochem Inc., San Diego, CA, USA) was injected in running buffer. At the end of the injection, running buffer was applied for 1000 s at 10 μL/s. 

### 3.9. SPRi Data Processing

The large amount of raw binding data produced by the SPRi was analyzed by Scrubber 2.0 software. The raw SPRi data was produced by SPRit software (Plexera Inc., Seattle, WA, USA), which annotated the p53 mutant array features, converted camera units into refractive index units, and performed background referencing to remove bulk refractive index effects. The SPRit software allowed the measurement of binding signal responses at various time points in units of refractive index. The data exported from SPRit software was transferred to CLAMP XP software to make it compatible with Scrubber 2.0 analysis. By using Scrubber software, the data were prepared for the global analysis by subtracting the average of the response over 20 s prior to the query protein injection and zeroing the time of injection in each experiment. The corrected binding data was then analyzed by direct curve fitting to a simple biomolecular interaction mechanism (1:1). These were fit to the association and dissociation phase sensor data in all the experiments. The error space for each of the parameters was assessed using statistical profiling (Rmax < 0.001). Each feature (e.g., p53 mutant proteins) was printed in duplicate on each array, and the experiment was performed in triplicate. In the case of wt p53, binding parameters from MDM2 titration studies were also included in the data processing (Supplementary Figure S7). Therefore, from 6 to 10 binding parameters (kon, koff, and KD) were generated for each feature. All binding values (kon, koff, and KD) that fell outside reasonable detection limits (i.e., KD above 1 µM or less than 1 nM and kon > 108 M^−1^s^−1^, or koff ≥ 1 s^−1^) or were affected by experimental processes such as bubbles, zone variation, etc., which could not be measured accurately, were eliminated from the statistical analysis.

If the overall computation of the values for a mutant fell outside instrument limits, no numerical result was reported. Instead, the value was listed as “<1 nM” or “>1 µM”, as appropriate. In all cases, we examined the sensorgrams to ensure that the binding curves were consistent with either “tight binding” or “weak binding” as implied by the out-of-range value reported. The statistical analysis was performed by MathLab v 7.8 (2009) software considering a normal distribution of each data set (kon, koff) per feature and per assay using median absolute deviation for a robust measure of statistical dispersion of the obtained data excluding values (kon, koff) outside the criteria (average ± 2 SD). Affinity constant (KD) was established as koff/kon values.

## 4. Conclusions

Our results described a method readily adaptable to a high-throughput format to measure the real-time interaction of a query protein with many full-length target proteins, without having the necessity to produce and purify the target proteins. This protein microarray fabrication method showed huge potential to be implemented for multiplexed SPRi biosensing measurements in both clinical and research applications.

One of the key advantages of our method is indicated by its ability to study full-length proteins, which has been challenging by conventional methods. For that, a novel NAPPA chemistry has been developed and optimized in order to adapt multiplex in situ protein expression suitable for SPRi. This novel chemistry is based on K-coil/E-coil interaction which allows capturing the in situ expressed proteins with high efficiency (high-affinity similar to streptavidin-biotin) and very close to the sensing surface, due to the small size of the tag which might increase the sensitivity. Interestingly, this chemistry is stable enough for use in affinity purification in protein chromatography [15]. In addition, this chemistry is rapid and simple (compare with previously described NAPPA chemistry), highly reproducible, stable, and robust. However, this chemistry could present a disadvantage for some protein interactions studies due to the steric hindrance from the proximity of captured protein to the surface. In this study, we included the GST protein as a spacer to avoid this limitation.

Furthermore, as the values of binding parameters obtained by SPRi are comparable with the ones obtained by conventional SPR, this novel method could be a suitable approach for determining relative quantitative binding parameters values amongst numerous proteins in a high-throughput format. There are numerous applications that would directly benefit from an early and rapid assessment of relative binding affinities amongst many candidates. However, in order to generate a more precise and acute understanding, these values should be subjected to further refinement by conducting extensive studies on lower throughput systems.

## Figures and Tables

**Table 1:** p53-MDM2 binding. Nutlin-3 response in vivo.

		KD	kon	koff	
Spot	Mutant ID	Log(Ratio KD Nutlin3/Non-Nutlin3	Log(Ratio kon Nutlin3/Non-Nutlin3)	Log(Ratio Nutlin3/Non-Nutlin3)	Nutlin Response
M29	Val157Phe	−1.060	0.0949	−0.962	Resistant
M38	His179Arg	−2.40	0.0714	−1.69	Resistant
M49	Tyr234Cys	−1.89	−1.18	−3.07	Resistant
	*Average*	−1.78	−0.123	−1.91	
M51	Asp281Asn	3.30	−3.74	−0.435	Intermediate
M2	Gly245Asp	1.44	−2.75	−1.31	Intermediate
	*Average*	2.73	−3.25	−0.0873	
M1	Gly245Ser	6.26	−5.49	−0.770	Sensitive
53WT	p53-WT	4.91	−4.63	−0.027	Sensitive
M39	His179Tyr	4.90	−4.17	0.740	Sensitive
M22	Arg280Thr	6.80	−4.33	−2.480	Sensitive
	*Average*	5.73	−4.66	−0.695	

## Data Availability

Data available at www.biodata.usal.es (accessed on 12 November 2021) repository. Ref: p53-Lab11.

## Supplementary Materials

The following are available online at https://www.mdpi.com/article/10.3390/ijms23010053/s1.
