# Supplementary material for: A Quantitative Systems Approach to Define Novel Effects of Tumour p53 Mutations on Binding Oncoprotein MDM2
Source: Int J Mol Sci. 2021 Dec 21;23(1):53. doi: 10.3390/ijms23010053 (PMC8744954; doi:10.3390/ijms23010053)
Supplement: Supplementary file 1 [file ijms-23-00053-s001.zip › ijms-1483913-supplementary 2.pdf]

SUPPLEMENTARY DATA

Supplementary Figure Legends

Figure S1: (A) The self-assembled monolayer (SAM) terminating with amines, activated esters, and polyethylene glycol were formed on gold surface. (B) The bare SAM was tested using profilometer, scanning electron microscopy (SEM) and atomic force microscopy (AFM). The SEM image demonstrated that SAMs formed uniformly on the gold coated surface. The height of the average monolayer was analyzed by AFM to be ~ 2 nm.

Figure S2: (A) The vector map of pANT7_GST_*E*-coil. (B) The pANT7_GST_*E*-coil sequence, where GST is highlighted in green color and *E*-coil in red color.

Figure S3: (A) An array of GST-*E*-coil tagged plasmid DNA was expressed using *in vitro* transcription and translation mix. The specificity of binding was demonstrated by injecting monoclonal antibodies (anti-p53, anti-Fos and anti-Jun) sequentially. Only p53, Fos and Jun protein spots exhibited binding, while no binding was observed in neighboring spots. (B) For ease of viewing, most of the non-binding sensorgrams have been removed and the protein identities of the individual colored sensorgrams are indicated.

Figure S4: Duplicate arrays of p53 mutants, printed with spatially separated duplicate features, were produced according to the NAPPA method. Purified full-length recombinant MDM2 protein was used as a query and the binding event of p53-MDM2 interaction was detected using NAPPA-SPR*i* in duplicate experiments performed on different days. The spot

to spot reproducibility on same chip as well as on the replicate chip is demonstrated for the indicated examples.

Figure S5: The protein-protein interaction of Jun-Fos assessed by NAPPA-SPR*i*. An array of proteins was produced according to the NAPPA method. The purified recombinant proteins Fos was used as query and the binding event of Jun-Fos interaction was detected using SPR*i* and fitted using Scrubber 2.0 software. The binding event of triplicate spots from two chips demonstrated the reproducibility in different experiments.

Figure S6: Representation of the tumor suppressor p53 protein with various known domains indicated as well as the locations of commonly noted clinical mutations used in our study.

Figure S7: Several representative individual sensorgrams that were fitted to a two-component model (association and dissociation) by Scrubber 2.0 to produce kinetic parameters. Orange line corresponds to the fit curve, green line corresponds to injection point starting the protein association, and blue line corresponds to the dissociation stage of the protein interaction after the stochastic steady state. Note that the arrival of binding query protein at one end of the array before the other end results in slightly different binding start times from one protein feature to the others. Full-length purified recombinant MDM2 in running buffer was added and then followed by running buffer alone at $t \approx 300s$.

SUPPLEMENTARY FIGURES

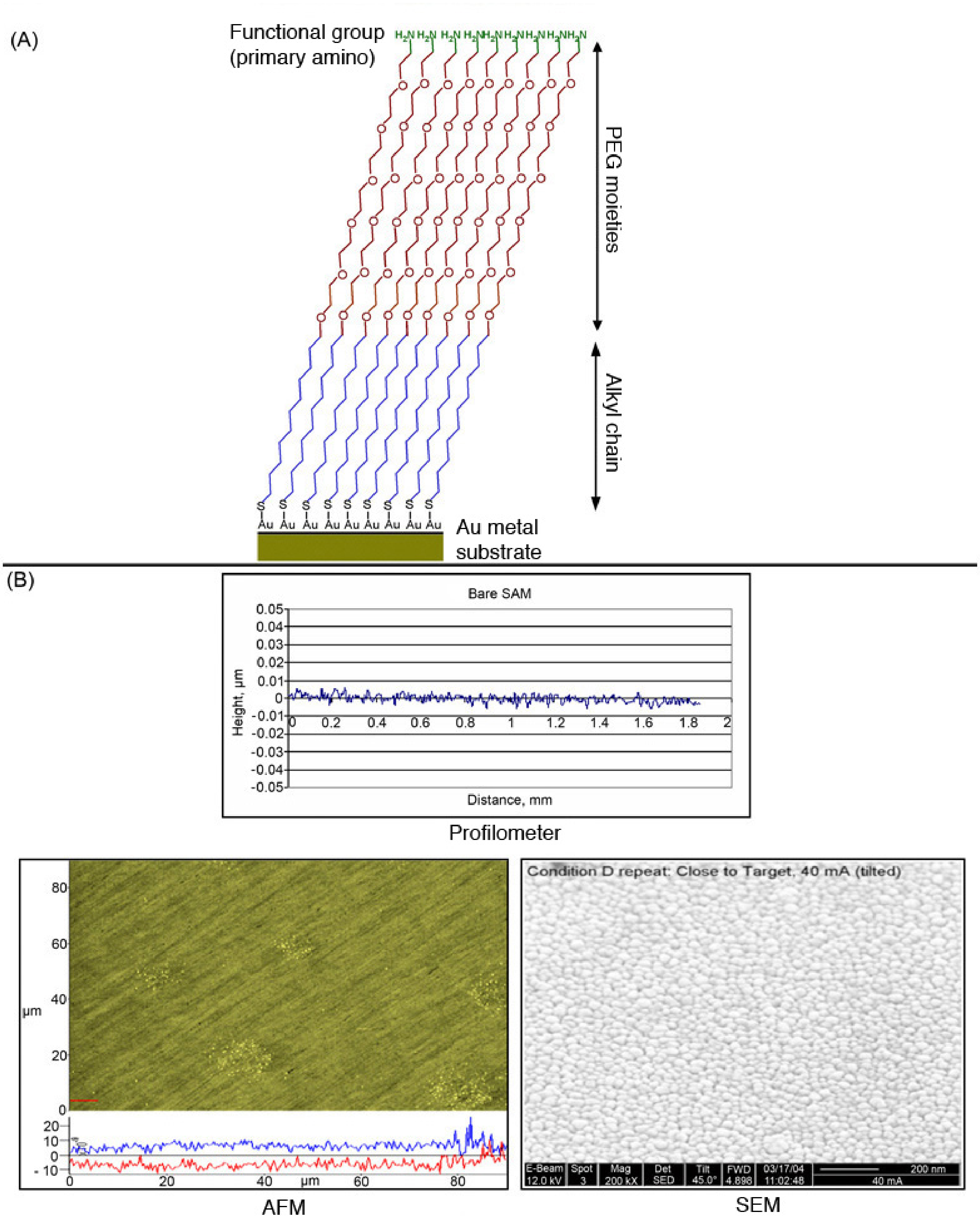
(A)
Functional group
(primary amino)
PEG moieties
Alkyl chain
Au metal
substrate
(B)
Bare SAM
Height, µm
Distance, mm
Profilometer
µm
AFM
Condition D repeat: Close to Target, 40 mA (tilted)
SEM

Figure S1

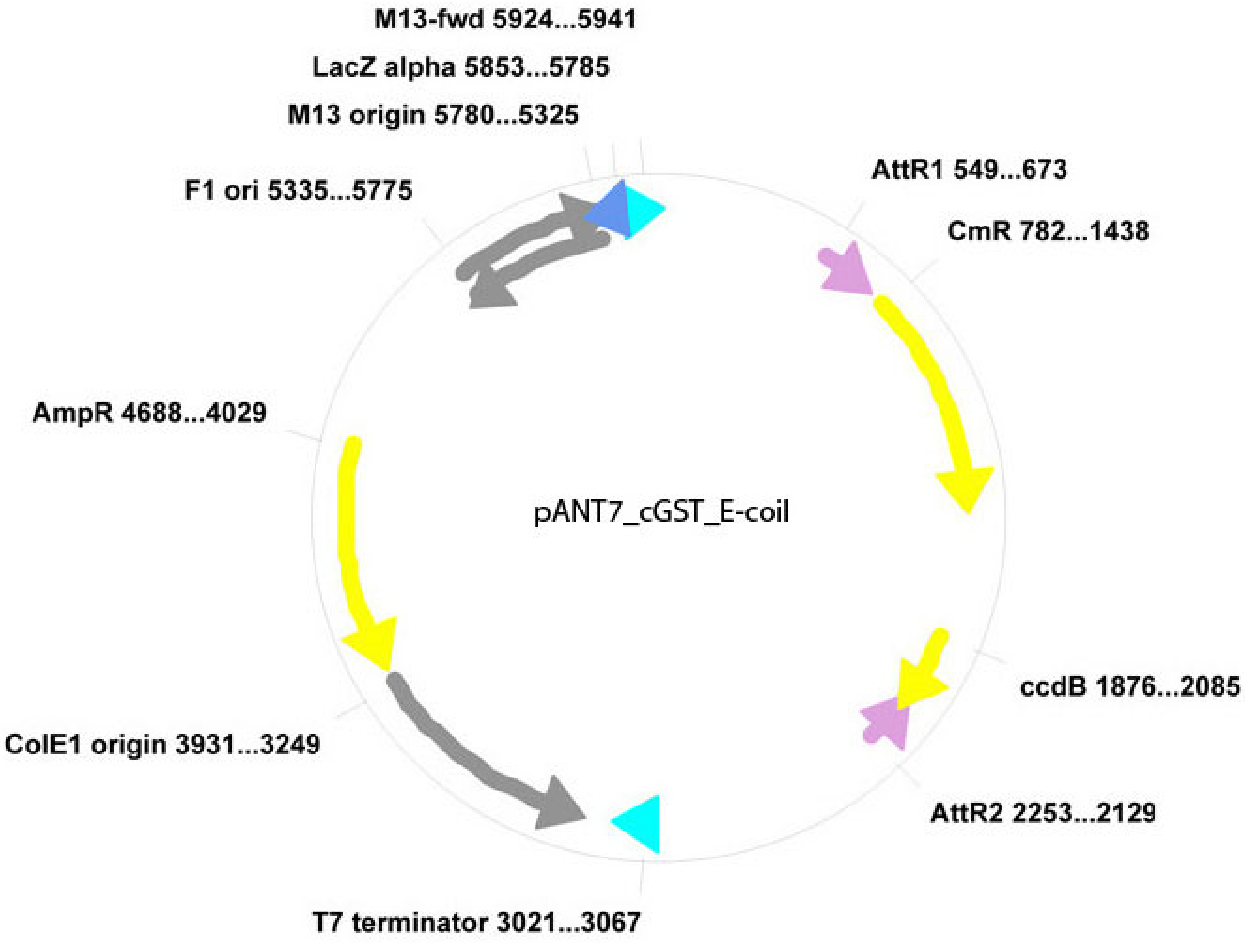
M13-fwd 5924...5941
LacZ alpha 5853...5785
M13 origin 5780...5325
F1 ori 5335...5775
AttR1 549...673
CmR 782...1438
AmpR 4688...4029
pANT7_cGST_E-coil
ccdB 1876...2085
ColE1 origin 3931...3249
AttR2 2253...2129
T7 terminator 3021...3067

Figure S2A

Figure S2B

pANT-GST-*E*-coil vector sequence

GGGCGAATTAATTCCGGTTATTTTCCACCATATTGCCGTCTTTTGGCAATGTGAGGGCCC
GGAAACCTGGCCCTGTCTTCTTGACGAGCATTCCTAGGGGTCTTTCCCCTCTCGCCAAAG
GAATGCAAGGTCTGTTGAATGTCGTGAAGGAAGCAGTTCCTCTGGAAGCTTCTTGAAGAC
AAACAACGTCTGTAGCGACCCTTTGCAGGCAGCGGAACCCCCCACCTGGCGACAGGTGCC
TCTGCGGCCAAAAGCCACGTGTATAAGATACACCTGCAAAGGCGGCACAACCCCAGTGCC
ACGTTGTGAGTTGGATAGTTGTGGAAAGAGTCAAATGGCTCACCTCAAGCGTATTCAACA
AGGGGCTGAAGGATGCCCAGAAGGTACCCCATTGTATGGGATCTGATCTGGGGCCTCGGT
GCACATGCTTTACATGTGTTTAGTCGAGGTTAAAAAACGTCTAGGCCCCCCGAACCACGG
GGACGTGGTTTTCCTTTGAAAAACACGATGATAAT**ATGGATCGGATCCGAATTCGAGCTC**
CGTCATCAACAAGTTTGTACAAAAAAGCTGAACGAGAAACGTAAAATGATATAAATATCA
ATATATTAAATTAGATTTTGCATAAAAAACAGACTACATAATACTGTAAAACACAACATA
TCCAGTCACTATGGCGGCCGCATTAGGCACCCCAGGCTTTACACTTTATGCTTCCGGCTC
GTATAATGTGTGGATTTTGAGTTAGGATCCGTCGAGATTTTCAGGAGCTAAGGAAGCTAA
AATGGAGAAAAAAATCACTGGATATACCACCGTTGATATATCCCAATGGCATCGTAAAGA
ACATTTTGAGGCATTTCAGTCAGTTGCTCAATGTACCTATAACCAGACCGTTCAGCTGGA
TATTACGGCCTTTTTAAAGACCGTAAAGAAAAATAAGCACAAGTTTTATCCGGCCTTTAT
TCACATTCTTGCCCGCCTGATGAATGCTCATCCGGAATTCCGTATGGCAATGAAAGACGG
TGAGCTGGTGATATGGGATAGTGTTCACCCTTGTTACACCGTTTTCCATGAGCAAACTGA
AACGTTTTCATCGCTCTGGAGTGAATACCACGACGATTTCCGGCAGTTTCTACACATATA
TTCGCAAGATGTGGCGTGTTACGGTGAAAACCTGGCCTATTTCCCTAAAGGGTTTATTGA
GAATATGTTTTTCGTCTCAGCCAATCCCTGGGTGAGTTTCACCAGTTTTGATTTAAACGT
GGCCAATATGGACAACTTCTTCGCCCCCGTTTTCACCATGGGCAAATATTATACGCAAGG
CGACAAGGTGCTGATGCCGCTGGCGATTCAGGTTCATCATGCCGTCTGTGATGGCTTCCA
TGTCGGCAGAATGCTTAATGAATTACAACAGTACTGCGATGAGTGGCAGGGCGGGGCGTA
AAGATCTGGATCCGGCTTACTAAAAGCCAGATAACAGTATGCGTATTTGCGCGCTGATTT
TTGCGGTATAAGAATATATACTGATATGTATACCCGAAGTATGTCAAAAAGAGGTGTGCT
ATGAAGCAGCGTATTACAGTGACAGTTGACAGCGACAGCTATCAGTTGCTCAAGGCATAT
ATGATGTCAATATCTCCGGTCTGGTAAGCACAACCATGCAGAATGAAGCCCGTCGTCTGC
GTGCCGAACGCTGGAAAGCGGAAAATCAGGAAGGGATGGCTGAGGTCGCCCGGTTTATTG
AAATGAACGGCTCTTTTGCTGACGAGAACAGGGACTGGTGAAATGCAGTTTAAGGTTTAC
ACCTATAAAAGAGAGAGCCGTTATCGTCTGTTTGTGGATGTACAGAGTGATATTATTGAC
ACGCCCGGGCGACGGATGGTGATCCCCCTGGCCAGTGCACGTCTGCTGTCAGATAAAGTC
TCCCGTGAACTTTACCCGGTGGTGCATATCGGGGATGAAAGCTGGCGCATGATGACCACC
GATATGGCCAGTGTGCCGGTCTCCGTTATCGGGGAAGAAGTGGCTGATCTCAGCCACCGC
GAAAATGACATCAAAAACGCCATTAACCTGATGTTCTGGGGAATATAAATGTCAGGCTCC
CTTATACACAGCCAGTCTGCAGGTCGACCATAGTGACTGGATATGTTGTGTTTTACAGTA
TTATGTAGTCTGTTTTTTATGCAAAATCTAATTTAATATATTGATATTTATATCATTTTA
CGTTTCTCGTTCAGCTTTCTTGTACAAAGTGGTTGAT**GACAAGCTTGCGGCCGCAC**
TCGAGCCTATACTAGGTTATTGGAAAATTAAGGGCCTTGTGCAACCCACTCGACTTCTT
TTGGAATATCTTGAAGAAAAATATGAAGAGCATTTGTATGAGCGCGATGAAGGTGATAAA
TGGCGAAACAAAAAGTTTGAATTGGGTTTGGAGTTTCCCAATCTTCCTTATTATATTGAT
GGTGATGTTAAATTAACACAGTCTATGGCCATCATACGTTATATAGCTGACAAGCACAAC
ATGTTGGGTGGTTGTCCAAAAGAGCGTGCAGAGATTTCAATGCTTGAAGGAGCGGTTTTG
GATATTAGATACGGTGTTTCGAGAATTGCATATAGTAAAGACTTTGAAACTCTCAAAGTT
GATTTTCTTAGCAAGCTACCTGAAATGCTGAAAATGTTCGAAGATCGTTTATGTCATAAA
ACATATTTAAATGGTGATCATGTAACCCATCCTGACTTCATGTTGTATGACGCTCTTGAT
GTTGTTTTATACATGGACCCAATGTGCCTGGATGCGTTCCCAAAATTAGTTTGTTTTAAA
AAACGTATTGAAGCTATCCCACAAATTGATAAGTACTTGAAATCCAGCAAGTATATAGCA
TGGCCTTTGCAGGGCTGGCAAGCCACGTTTGGTGGTGGCGACCATCCTCCAAAATCGGAT
CTGGTTCCGCGTCTCGA
GGGTGGAGGGCTTGAAGTTTCTGCTCTTGAAAAAGAAGTCTCCGCGTTGGAGAAAGAGGTGAGCGCGCTGG
AGAAAGAAGTTAGCGCCTTGGAGAAGGAAGTTTCTGCACTAGAGAAGTGAGCTAGCGGATCTGACTGAAAA
AAAAAAAAAAAAAAAAAAAAAAAAAAAGTTTAAACACTAGTCCGCTGAGCAATAACTAGCATAACCCCTTGG
GGCCTCTAAACGGGTCTTGAGGGGTTTTTTGCTGAAAGGAGGAACTATATCCGGGCTTCCTCGCTCACTGA
CTCGCTGCGCTCGGTCGTTCGGCTGCGGCGAGCGGTATCAGCTCACTCAAAGGCGGTAATACGGTTATCCA
CAGAATCAGGGGATAACGCAGGAAAGAACATGTGAGCAAAAGGCCAGCAAAAGGCCAGGAACCGTAAAAAG
GCCGCGTTGCTGGCGTTTTTCCATAGGCTCCGCCCCCCTGACGAGCATCACAAAAATCGACGCTCAAGTCA
GAGGTGGCGAAACCCGACAGGACTATAAAGATACCAGGCGTTTCCCCCTGGAAGCTCCCTCGTGCGCTCTC
CTGTTCCGACCCTGCCGCTTACCGGATACCTGTCCGCCTTTCTCCCTTCGGGAAGCGTGGCGCTTTCTCAT

```
AGCTCACGCTGTAGGTATCTCAGTTCGGTGTAGGTCGTTCGCTCCAAGCTGGGCTGTGTGCACGAACCCCC
CGTTCAGCCCGACCGCTGCGCCTTATCCGGTAACTATCGTCTTGAGTCCAACCCGGTAAGACACGACTTAT
CGCCACTGGCAGCAGCCACTGGTAACAGGATTAGCAGAGCGAGGTATGTAGGCGGTGCTACAGAGTTCTTG
AAGTGGTGGCCTAACTACGGCTACACTAGAAGGACAGTATTTGGTATCTGCGCTCTGCTGAAGCCAGTTAC
CTTCGGAAAAAGAGTTGGTAGCTCTTGATCCGGCAAACAAACCACCGCTGGTAGCGGTGGTTTTTTTGTTT
GCAAGCAGCAGATTACGCGCAGAAAAAAAGGATCTCAAGAAGATCCTTTGATCTTTTCTACGGGGTCTGAC
GCTCAGTGGAACGAAAACTCACGTTAAGGGATTTTGGTCATGAGATTATCAAAAAGGATCTTCACCTAGAT
CCTTTTAAATTAAAAATGAAGTTTTAAATCAATCTAAAGTATATATGAGTAAACTTGGTCTGACAGTTACC
AATGCTTAATCAGTGAGGCACCTATCTCAGCGATCTGTCTATTTCGTTCATCCATAGTTGCCTGACTCCCC
GTCGTGTAGATAACTACGATACGGGAGGGCTTACCATCTGGCCCCAGTGCTGCAATGATACCGCGAGACCC
ACGCTCACCGGCTCCAGATTTATCAGCAATAAACCAGCCAGCCGGAAGGGCCGAGCGCAGAAGTGGTCCTG
CAACTTTATCCGCCTCCATCCAGTCTATTAATTGTTGCCGGGAAGCTAGAGTAAGTAGTTCGCCAGTTAAT
AGTTTGCGCAACGTTGTTGCCATTGCTACAGGCATCGTGGTGTCACGCTCGTCGTTTGGTATGGCTTCATT
CAGCTCCGGTTCCCAACGATCAAGGCGAGTTACATGATCCCCCATGTTGTGCAAAAAAGCGGTTAGCTCCT
TCGGTCCTCCGATCGTTGTCAGAAGTAAGTTGGCCGCAGTGTTATCACTCATGGTTATGGCAGCACTGCAT
AATTCTCTTACTGTCATGCCATCCGTAAGATGCTTTTCTGTGACTGGTGAGTACTCAACCAAGTCATTCTG
AGAATAGTGTATGCGGCGACCGAGTTGCTCTTGCCCGGCGTCAATACGGGATAATACCGCGCCACATAGCA
GAACTTTAAAAGTGCTCATCATTGGAAAACGTTCTTCGGGGCGAAAACTCTCAAGGATCTTACCGCTGTTG
AGATCCAGTTCGATGTAACCCACTCGTGCACCCAACTGATCTTCAGCATCTTTTACTTTCACCAGCGTTTC
TGGGTGAGCAAAAACAGGAAGGCAAAATGCCGCAAAAAAGGGAATAAGGGCGACACGGAAATGTTGAATAC
TCATACTCTTCCTTTTTCAATATTATTGAAGCATTTATCAGGGTTATTGTCTCATGAGCGGATACATATTT
GAATGTATTTAGAAAAATAAACAAATAGGGGTTCCGCGCACATTTCCCCGAAAAGTGCCACCTGACGTCTA
AGAAACCATTATTATCATGACATTAACCTATAAAAATAGGCGTATCACGAGGCCCTTTCGTCTCGCGCGTT
TCGGTGATGACGGTGAAAACCTCTGACACATGCAGCTCCCGGAGACGGTCACAGCTTGTCTGTAAGCGGAT
GCCGGGAGCAGACAAGCCCGTCAGGGCGCGTCAGCGGGTGTTGGCGGGTGTCGGGGCTGGCTTAACTATGC
GGCATCAGAGCAGATTGTACTGAGAGTGCACCATATATGCGGTGTGAAATACCGCACAGATGCGTAAGGAG
AAAATACCGCATCAGGAAATTGTAAACGTTAATATTTTGTTAAAATTCGCGTTAAATTTTTGTTAAATCAG
CTCATTTTTTAACCAATAGGCCGAAATCGGCAAAATCCCTTATAAATCAAAAGAATAGACCGAGATAGGGT
TGAGTGTTGTTCCAGTTTGGAACAAGAGTCCACTATTAAAGAACGTGGACTCCAACGTCAAAGGGCGAAAA
ACCGTCTATCAGGGCGATGGCCCACTACGTGAACCATCACCCTAATCAAGTTTTTTGGGGTCGAGGTGCCG
TAAAGCACTAAATCGGAACCCTAAAGGGAGCCCCCGATTTAGAGCTTGACGGGGAAAGCCGGCGAACGTGG
CGAGAAAGGAAGGGAAGAAAGCGAAAGGAGCGGGCGCTAGGGCGCTGGCAAGTGTAGCGGTCACGCTGCGC
GTAACCACCACACCCGCCGCGCTTAATGCGCCGCTACAGGGCGCGTCGCGCCATTCGCCATTCAGGCTGCG
CAACTGTTGGGAAGGGCGATCGGTGCGGGCCTCTTCGCTATTACGCCAGCTGGCGAAAGGGGGATGTGCTG
CAAGGCGATTAAGTTGGGTAACGCCAGGGTTTTCCCAGTCACGACGTTGTAAAACGACGGCCAGTGAATTG
TAATACGACTCACTATA
```

Green-GST
Red – *E*-coil
Blue – Death Cassette

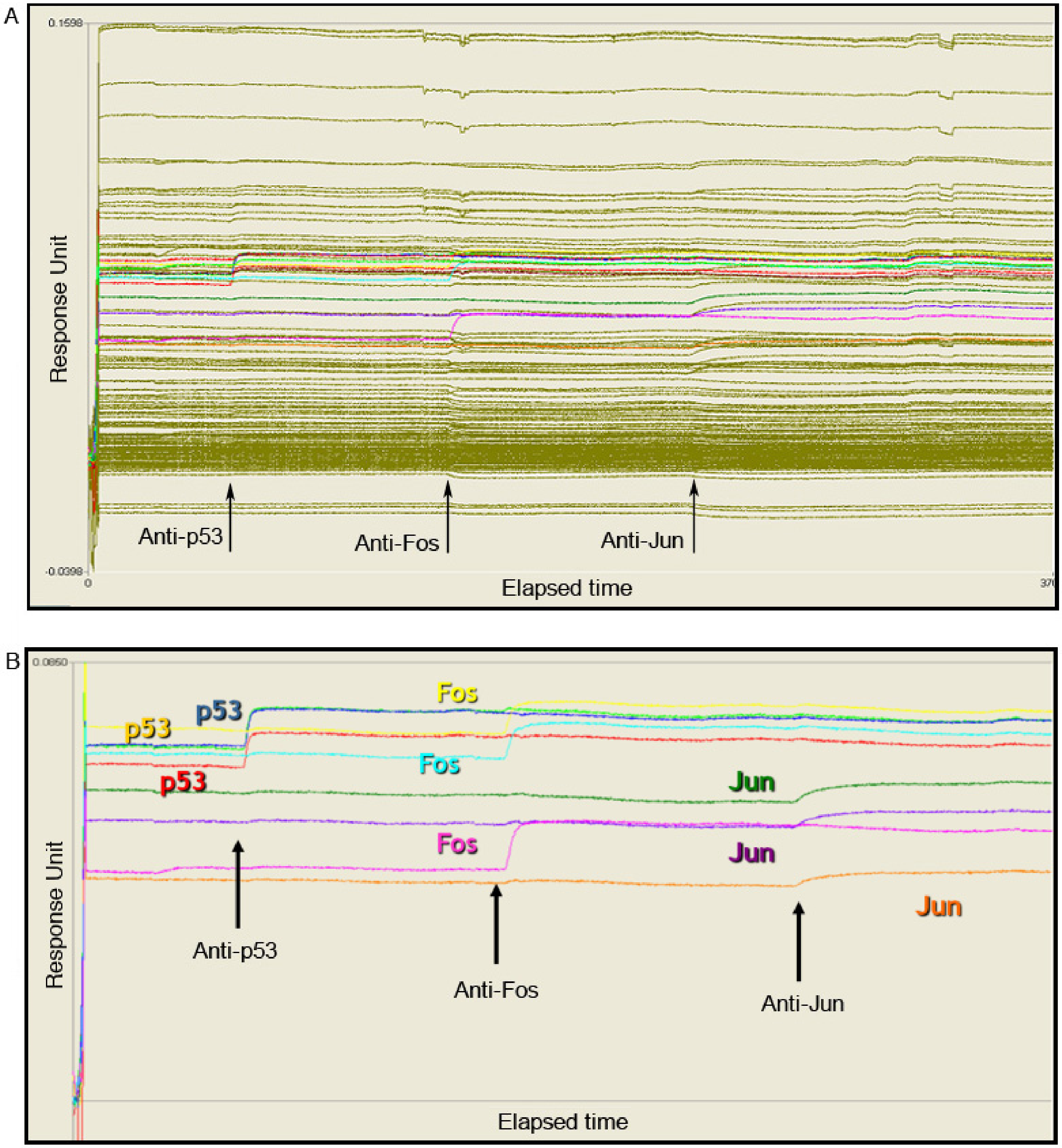
A
0.1598
-0.0398
0
Response Unit
Anti-p53
Anti-Fos
Anti-Jun
Elapsed time
B
0.0850
Response Unit
p53
p53
p53
Fos
Fos
Fos
Jun
Jun
Jun
Anti-p53
Anti-Fos
Anti-Jun
Elapsed time

Figure S3

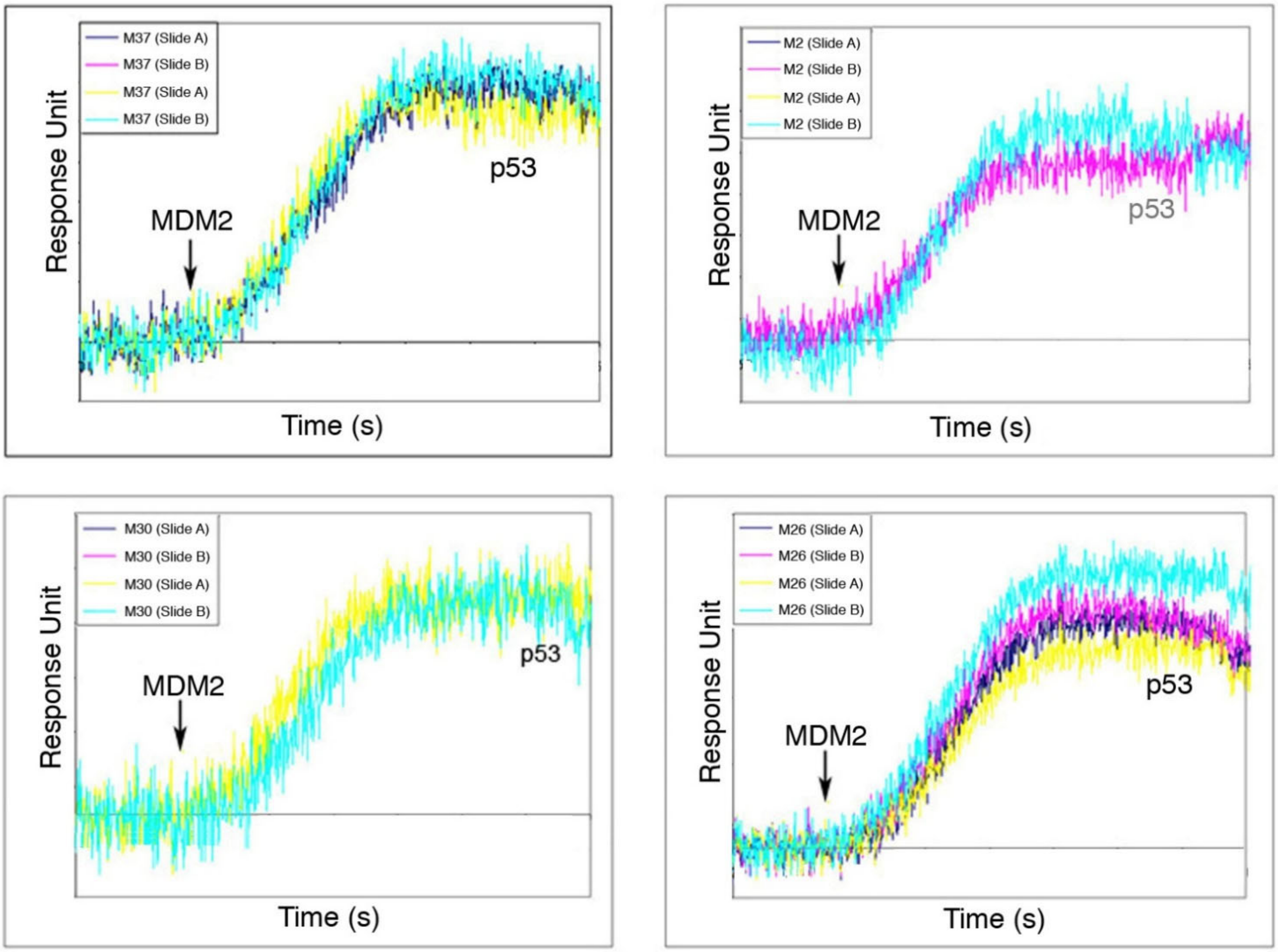
M37 (Slide A)
M37 (Slide B)
M37 (Slide A)
M37 (Slide B)
Response Unit
MDM2
p53
Time (s)
M2 (Slide A)
M2 (Slide B)
M2 (Slide A)
M2 (Slide B)
Response Unit
MDM2
p53
Time (s)
M30 (Slide A)
M30 (Slide B)
M30 (Slide A)
M30 (Slide B)
Response Unit
MDM2
p53
Time (s)
M26 (Slide A)
M26 (Slide B)
M26 (Slide A)
M26 (Slide B)
Response Unit
MDM2
p53
Time (s)

Figure S4

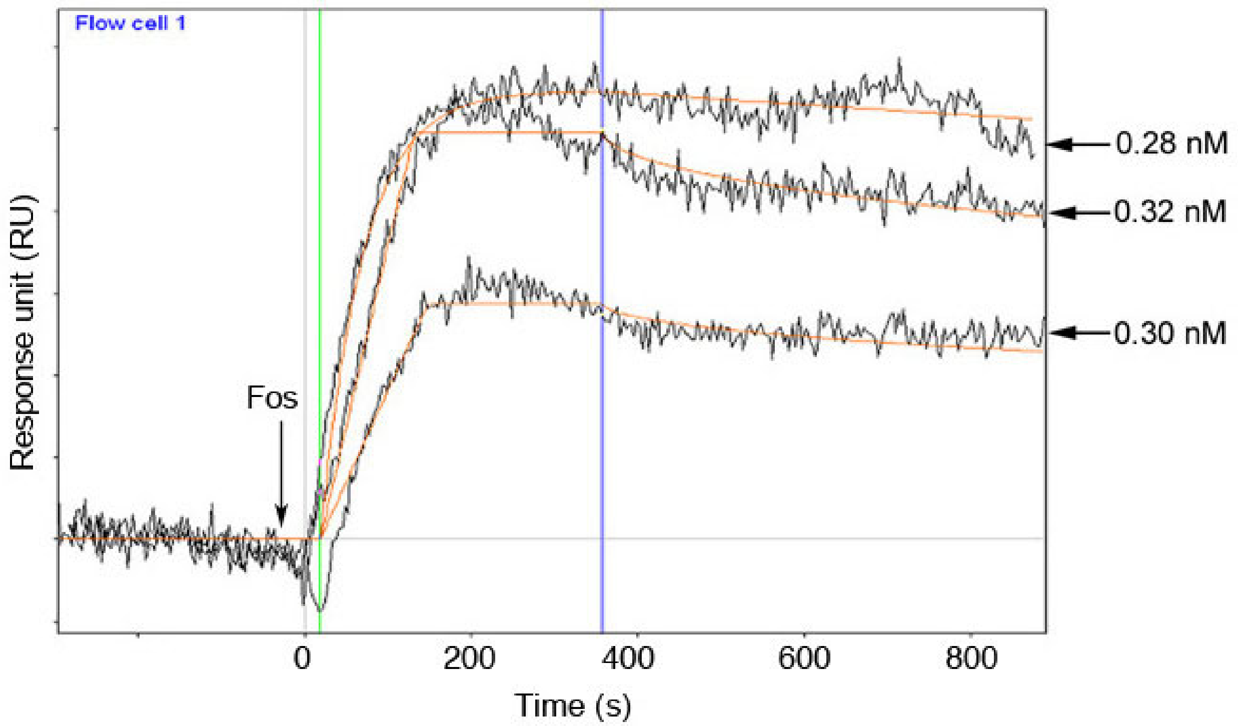
Flow cell 1
Response unit (RU)
Fos
0
200
400
600
800
Time (s)
0.28 nM
0.32 nM
0.30 nM

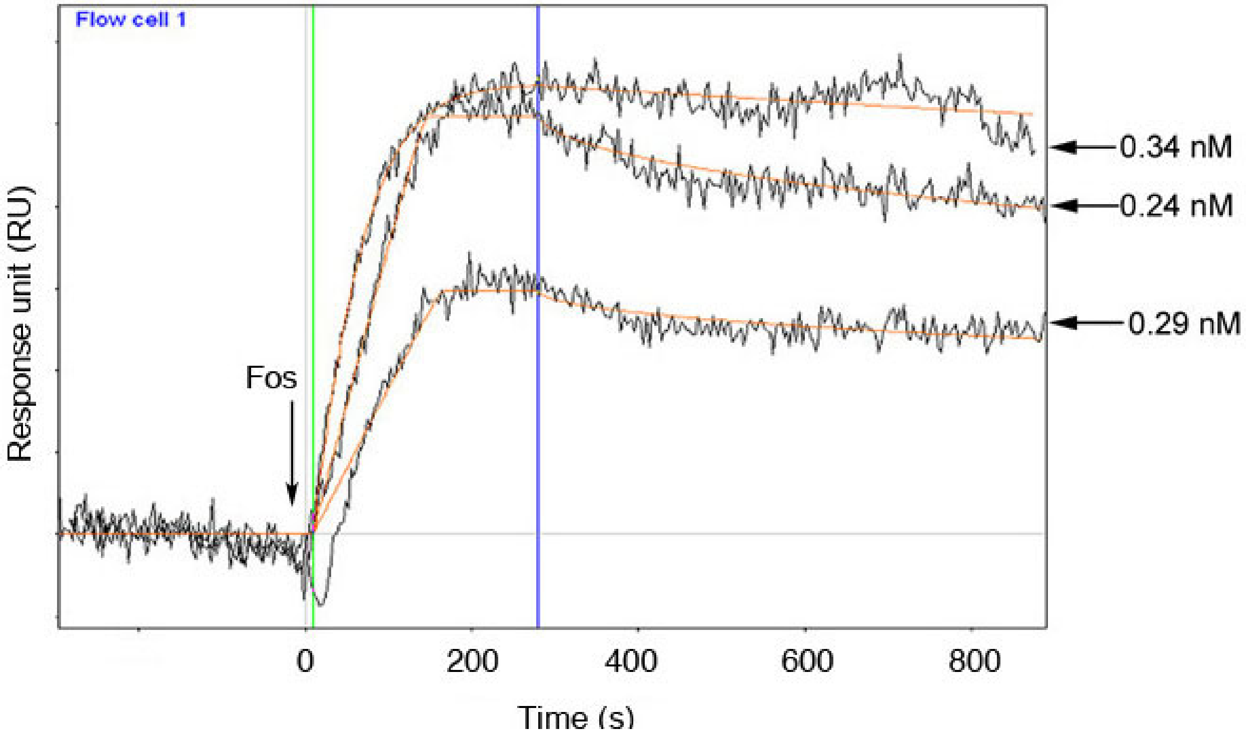
Flow cell 1
Response unit (RU)
Fos
0
200
400
600
800
Time (s)
0.34 nM
0.24 nM
0.29 nM

Figure S5

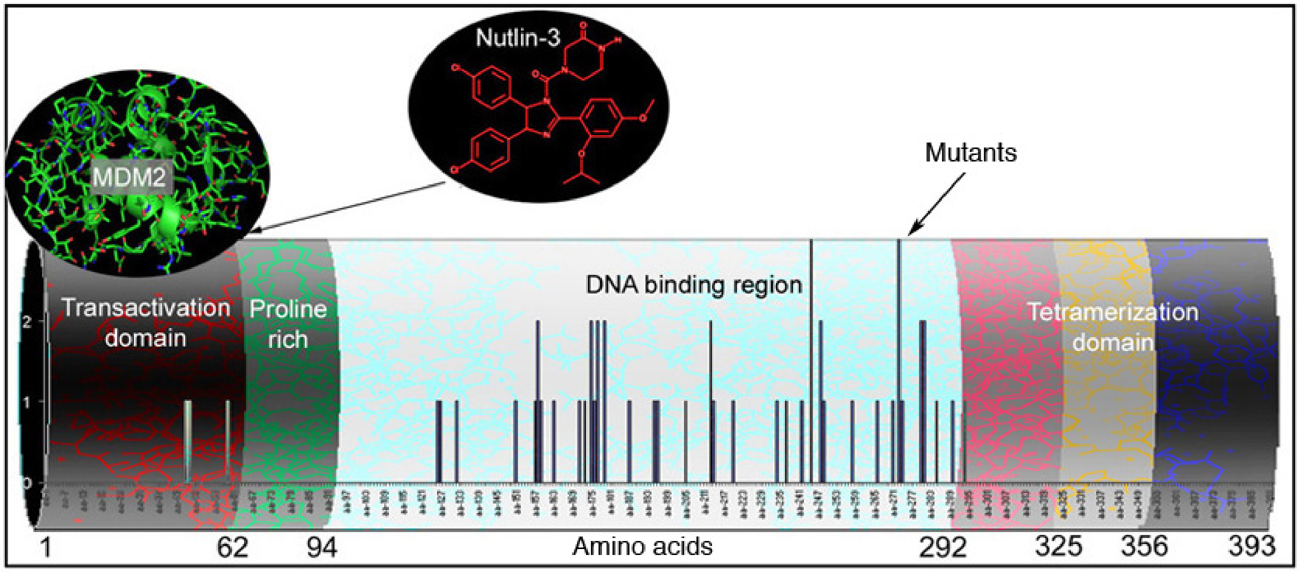
Nutlin-3
MDM2
Mutants
DNA binding region
Transactivation domain
Proline rich
Tetramerization domain
1
62
94
Amino acids
292
325
356
393

Figure S6

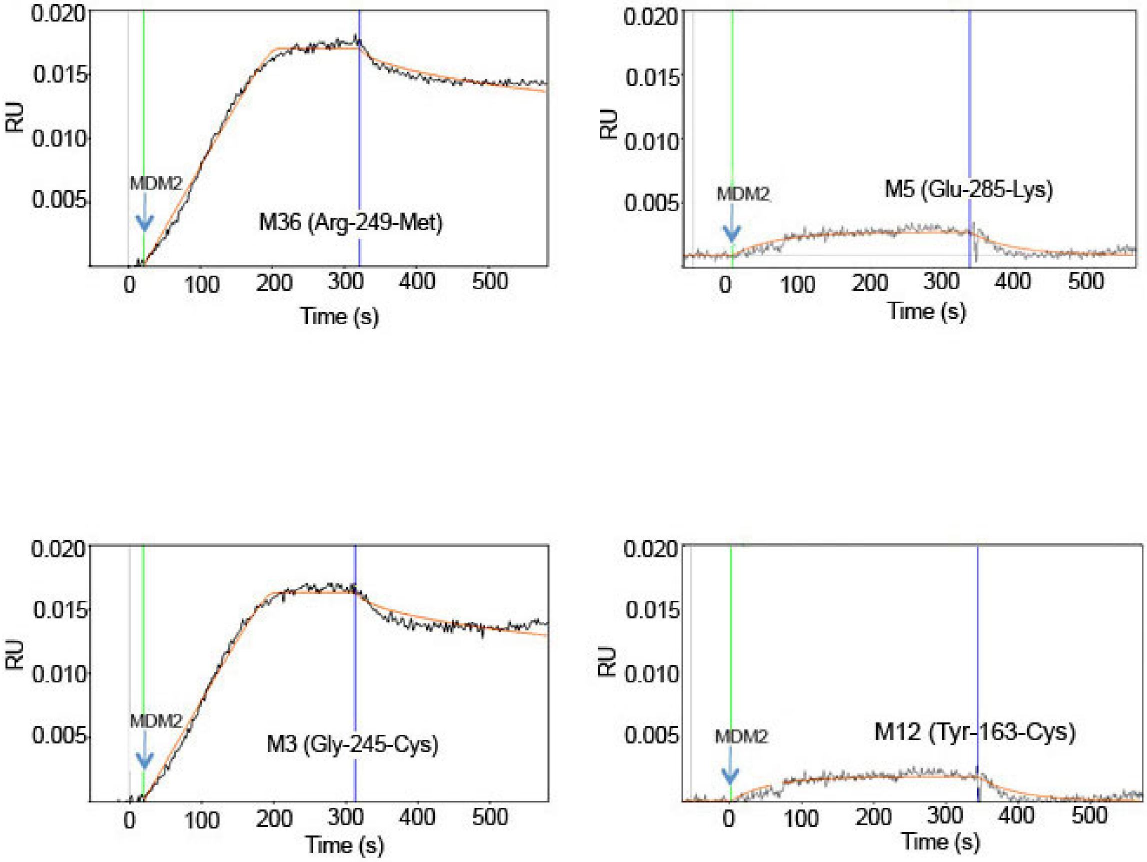
0.020
0.015
0.010
0.005
RU
MDM2
M36 (Arg-249-Met)
0
100
200
300
400
500
Time (s)
M5 (Glu-285-Lys)
M3 (Gly-245-Cys)
M12 (Tyr-163-Cys)

Fig. S-7

SUPPLEMENTARY TABLE S1

Quantitative measurement of the effect of Nutlin-3 binding on p53-MDM2 interaction

		(-) Nutlin3						(+)-Nutlin3					
Mutant	Mutant ID	kon average	kon SE	koff average	koff SE	KD average	KD SE	kon average	kon SE	koff	koff SE	KD average	KD SE
Arg213Stop	**M13**	1.88E+06	5.36E+05	2.45E-01	5.39E-03	1.30E-07	3.73E-09	2.85E+06	1.45E+03	3.18E-02	2.20E-02	1.12E-08	7.72E-11
Arg249Met	**M36**	1.76E+06	6.68E+05	8.11E-01	1.25E-03	4.60E-07	2.45E-09	1.17E+03	7.09E+02	4.54E-02	3.68E-02	3.88E-05	5.49E-07
p53-WT	**p53-WT**	2.23E+06	5.83E+05	1.90E-01	9.73E-04	8.52E-08	2.24E-09	5.20E+01	2.36E+02	3.57E-01	2.52E-01	6.86E-03	4.88E-04
Gly245Cys	**M3**	1.08E+06	3.50E+05	1.39E-01	1.78E-05	1.29E-07	4.21E-10	1.84E+06	8.16E+05	2.06E-02	1.06E-02	1.12E-08	1.07E-10
His179Arg	**M38**	6.98E+05	9.22E+04	1.29E-01	1.55E-03	1.85E-07	2.46E-09	3.61E+06	1.10E+06	2.63E-02	1.01E-02	7.27E-09	5.01E-11
Arg273Leu	**M18**	1.50E+06	3.33E+05	1.19E-01	1.17E-04	7.92E-08	1.76E-09	4.08E+06	5.01E+05	1.80E-02	7.91E-03	4.41E-09	2.48E-11
Tyr126Cys	**M27**	6.59E+05	2.05E+05	1.67E-01	1.10E-04	2.53E-07	7.87E-09	1.62E+06	2.96E+05	1.38E-02	6.25E-03	8.52E-09	5.41E-11
Glu285Lys	**M5**	1.20E+06	4.91E+05	1.67E-01	2.25E-04	1.39E-07	5.67E-09	1.45E+06	6.74E+05	8.95E-03	3.61E-03	6.15E-09	5.34E-11
Val157Phe	**M29**	1.33E+06	4.91E+05	1.67E-01	1.52E-03	1.26E-07	4.67E-09	1.65E+06	7.39E+05	1.83E-01	1.53E-01	1.11E-07	1.42E-09
Pro177Leu	**M40**	1.04E+06	1.66E+05	1.95E-01	4.94E-04	1.88E-07	3.01E-09	9.17E+03	6.13E+03	1.70E-02	7.31E-03	1.86E-06	2.04E-08
Glu258Lys	**M20**	6.03E+05	1.51E+05	4.27E-03	2.47E-03	7.08E-09	5.88E-11	1.93E+06	8.59E+04	1.08E-02	4.49E-03	5.60E-09	2.57E-11
Arg290His	**M46**	2.81E+06	1.44E+06	2.21E-01	6.91E-02	7.89E-08	6.52E-10	1.48E+06	7.97E+05	9.19E-03	5.73E-03	6.23E-09	7.25E-11
Glu171Stop	**M7**	8.14E+05	2.51E+04	8.48E-03	1.02E-04	1.04E-08	4.47E-12	1.04E+06	3.41E+05	6.85E-03	2.69E-03	6.58E-09	4.74E-11
Arg158Leu	**M31**	1.21E+06	2.92E+05	3.15E-01	9.62E-05	2.61E-07	6.30E-10	2.78E+06	3.67E+05	8.43E-03	3.11E-03	3.03E-09	1.52E-11
His214Arg	**M43**					<1nM		1.29E+06	6.45E+05	6.16E-03	2.96E-03	4.77E-09	4.67E-11
Arg280Thr	**M22**	5.54E+05	7.02E+04	1.88E-03	0.00E+00	3.39E-09	4.31E-13	2.58E+01	8.67E+04	5.73E-01	5.24E-01	2.23E-02	2.78E-03
Tyr234Cys	**M48**	3.81E+05	9.16E+04	1.67E-01	2.10E-04	4.38E-07	1.06E-09	9.09E+04	4.94E+04	5.27E-03	2.37E-03	5.80E-08	5.76E-10
Gly266Glu	**M9**	6.86E+05	1.09E+05	1.01E-02	9.25E-05	1.48E-08	2.47E-11			> 1s-1			
Arg175Cys	**M33**					<1nM		3.33E+02	2.36E+02	1.59E-02	9.22E-03	4.77E-05	6.14E-07
Gly187Ser	**M24**	1.28E+06	4.37E+05	7.34E-02	1.06E-04	5.72E-08	1.96E-10			> 1s-1			
Arg273His	**M50**					<1nM		7.21E+02	2.57E+02	8.32E-03	3.90E-03	1.15E-05	9.52E-08
Arg280Lys	**M11**	1.33E+06	3.01E+05	5.88E-01	1.64E-03	4.41E-07	1.01E-09	1.23E+06	7.49E+05	1.26E-02	6.21E-03	1.03E-08	1.13E-10
Val173Gly	**M34**	2.93E+06	1.75E+06	3.94E-01	1.25E-03	1.34E-07	8.03E-10	1.69E+05	1.38E+05	6.73E-03	2.95E-03	3.98E-08	4.99E-10
Glu271Lys	**M52**	2.04E+06	2.06E+05	2.15E-01	8.63E-03	1.05E-07	1.49E-10	5.00E+02	2.36E+02	4.59E-03	2.37E-03	9.17E-06	9.06E-08
Gly245Ser	**M1**	3.71E+06	1.49E+06	6.93E-02	3.71E-03	1.87E-08	8.53E-11	1.19E+01	3.82E+05	4.04E-01	3.30E+05	3.38E-02	3.85E-03
Tyr163Cys	**M12**					<1nM		1.82E+06	1.07E+04	> 1s-1			
Pro177Ser	**M35**					<1nM		3.82E+03	2.74E+03	> 1s-1			
Arg213Leu	**M15**	1.14E+06	2.49E+05	7.21E-01	1.49E-03	6.30E-07	1.38E-09					> 1µM	
Arg175His	**M25**	1.37E+06	3.19E+05	5.56E-01	2.95E-04	4.05E-07	9.45E-10					> 1µM	
Gly245Asp	**M2**	3.39E+05	1.61E+05	5.05E-02	3.59E-04	1.49E-07	7.18E-10	6.00E+02	2.24E+02	2.47E-03	1.11E-03	4.11E-06	3.37E-08
Met237Ile	**M14**	2.68E+06	1.15E+06	1.70E-01	2.00E-03	6.37E-08	2.82E-10	1.97E+05	6.99E+03	3.91E-03	2.00E-03	1.98E-08	1.08E-10
Tyr220Cys	**M37**					<1nM		5.00E+02	2.36E+02	3.38E-03	1.49E-03	6.75E-06	6.16E-08
Arg273Cys	**M17**					<1nM		3.33E+05	2.27E+05	1.67E-03	1.21E+05	5.01E-09	3.64E-03
Lys132Arg	**M26**	1.25E+06	4.05E+05	2.29E-01	4.34E-04	1.84E-07	6.00E-10	1.29E+05	7.28E+04	1.67E-03	1.11E-03	1.30E-08	1.59E-10
Pro47Ser	**M4**	2.04E+06	2.81E+05	1.02E-01	6.36E-06	4.99E-08	6.89E-11	3.06E+05	2.55E+05	> 1s-1			
Arg158His	**M28**					<1nM						> 1µM	
His179Tyr	**M39**	1.14E+06	4.05E+05	4.11E-02	1.16E-04	3.59E-08	1.28E-10	7.81E+01	3.03E+02	2.25E-01	1.84E-01	2.88E-03	2.36E-03
Val274Phe	**M19**	1.00E+03	0.00E+00	1.35E-03	3.03E-04	1.35E-06	3.03E-09	6.67E+02	2.11E+02	1.74E-03	1.05E-03	2.61E-06	2.39E-08
Ser127Phe	**M45**	3.93E+06	1.61E+06	2.18E-01	1.86E-03	5.54E-08	2.32E-10	1.33E+05	6.05E+02	5.00E-04	2.36E-04	3.76E-09	1.79E-11
Ser46Pro	**M6**	3.57E+06	1.25E+06	1.02E-02	4.12E-03	2.85E-09	2.15E-11	3.89E+05	3.37E+04	> 1s-1			
Ala159Pro	**M30**					<1nM						> 1µM	
Gly244Cys	**M41**					<1nM		1.15E+04	9.10E+03	2.35E-03	8.93E-04	2.03E-07	2.37E-09
Arg196Stop	**M21**	2.08E+06	8.79E+05	3.37E-02	3.38E-04	1.62E-08	7.04E-11					> 1µM	
Ile195Thr	**M47**					<1nM		7.50E+02	2.04E+02	2.53E-04	2.03E-04	3.37E-07	3.63E-09
Asp281His	**M8**					<1nM		7.50E+02	2.24E+02	3.33E-04	2.36E-04	4.44E-07	4.47E-09
Cys176Phe	**M32**					<1nM						> 1µM	
Pro151Ser	**M23**	2.03E+06	2.82E+05	5.22E-03	3.36E-03	2.58E-09	2.02E-11					> 1µM	
Tyr234Cys	**M49**	3.39E+06	1.02E+06	5.88E-01	6.13E-02	1.74E-07	7.04E-10	2.25E+05	2.65E+04	5.00E-04	2.36E-04	2.23E-09	1.31E-11
Tyr205Cys	**M10**	1.64E+06	1.04E+05	3.37E-02	3.54E-05	2.06E-08	1.32E-11	2.77E+03	1.86E+03	7.50E-04	2.04E-04	2.70E-07	2.55E-09
Asp281Asn	**M51**	3.29E+05	1.01E+06	1.36E-03	2.13E-04	4.14E-09	1.92E-12	6.00E+02	1.00E+01	5.00E-04	2.36E-04	8.33E-07	3.93E-09

Footnote – KD (Dissociation Constant), k_{on} (On rate), k_{off} (Off rate), SE (Standard error)

<u>**SUPPLEMENTARY TABLE S2**</u>

Quantitative measurement of the effect of Nutlin-3 binding on p53- MDM2 interaction

		KD	kon	koff
	**Mutant ID**	**Log10 KD/KD-N**	**Log10(kon/kon-N)**	**Log10(kon/kon-N)**
**M13**	Arg213Stop	-14.23	0.18	-1.89
**M36**	Arg249Met	-10.66	-3.18	-1.40
**p53-WT-1**	p53-WT	-4.90	4.63	-0.27
**M3**	Gly245Cys	-13.98	0.23	-0.83
**M38**	His179Arg	-13.98	0.71	-1.69
**M18**	Arg273Leu	-14.53	0.44	-1.82
**M27**	Tyr126Cys	-13.89	0.39	-2.08
**M5**	Glu285Lys	-14.29	0.08	-2.27
**M29**	Val157Phe	-13.08	0.09	-0.96
**M40**	Pro177Leu	-11.75	-2.05	-2.06
**M20**	Glu258Lys	-14.03	0.51	0.40
**M46**	Arg290His	-14.65	-0.28	-1.38
**M7**	Glu171Stop	-14.09	0.11	-0.09
**M31**	Arg158Leu	-14.60	0.36	-1.57
**M22**	Arg280Thr	-6.81	4.33	-2.48
**M48**	Tyr234Cys	-12.82	-0.62	-1.50
**M11**	Arg280Lys	-14.11	-0.04	-1.67
**M34**	Val173Gly	-13.87	-1.24	-1.77
**M52**	Glu271Lys	-11.35	-3.61	-1.67
**M1**	Gly245Ser	-6.25	5.49	-0.77
**M2**	Gly245Asp	-10.92	-2.75	-1.31
**M14**	Met237Ile	-14.13	-1.13	-1.64
**M26**	Lys132Arg	-13.98	-0.99	-2.14
**M39**	His179Tyr	-4.90	4.17	0.74
**M19**	Val274Phe	-8.58	-0.18	0.11
**M45**	Ser127Phe	-15.02	-1.47	-2.64
**M49**	Tyr234Cys	-15.18	-1.18	-3.07
**M10**	Tyr205Cys	-12.78	-2.77	-1.65
**M51**	Asp281Asn	-11.60	-2.74	-0.44

Note - mutants M43, M33, M50, M12, M35, M15, M25, M37, M17, M28, M30, M41, M47, M8, M35, M24, M12, M4, M6, M9, and M32 had at least one parameter out of range and hence not included in this table.

SUPPLEMENTARY TABLE S3
Effect of Nutlin-3 binding on cell behavior

Spot	Mutant ID	Nutlin Response	Cell-line info	References
M18	Arg273Leu	Resistant	HT 29 (colorectal cancer)	S1-10
M29	Val157Phe	Resistant	Hs578T (breast cancer)	S1-8, S12-13
M38	His179Arg	Resistant	SK-OV-3 (ovarian cancer)/ KM12 (Colorectal cancer/ KYSE-450 (esophageal carcinoma)	S1-10, S14-19
M50	Arg273His	Resistant	SW480 (colorectal cancer)/ OVCAR-3 ovarian cancer)	S1-10,S 14-15
M2	Gly245Asp	Intermediate	GLY, LS-1034 (colorectal cancer)/LY7 (B-cell lymphoma)	S1-10, S17,S20-22
M51	Arg281Asn	Intermediate	Nutlin3 >10μM	S1-10
p53WT	p53-WT	Sensitive		
M1	Gly245Ser	Sensitive	SU86.86 (Pancreatic cancer)/ NMB (Neuroblastoma)/ C10, CBS, CC07 (Colorectal Cancer)/KOPM30 (B-acute lymphoblasctic leukemia)/ RIT2 (Osteosarcoma)/HSC39 (melanoma)/D-336MG, D-423MG, D-566MG (Brain tumor)	S1-10, 25-31
M22	Arg280Thr	Sensitive	BL-17 (bladder carcinoma)	S1-8, 11
M39	His179Tyr	Sensitive	SK-OV-3 (ovarian cancer)/ KM12 (Colorectal cancer/ SK-PC-3 (Pancreatic cancer)	S1-10

* The number refers to the citations for supplementary references.

Supplementary References

S1. Hjorstberg, L., Rubio-Nevado, J .M., Hamroun, D., Claustre, M., Beroud, C., Soussi, T., The p53 Mutation HandBook http://p53.free.fr/Database/p53_database.html (2008).

S2. Carvajal, D., Tovar,C., Yang,H., Vu, B.T., Heimbrook, D.C., Vassilev,L.T., Activation of p53 by MDM2 Antagonists Can Protect Proliferating Cells from Mitotic Inhibitors, *Cancer Res* **65**, 1918-1924 (2005).

S3. Hu, B., Gilkes, D.M., Farooqi, B., Sebti, S., Chen,J. MDMX overexpression prevents p53 activation by the Mdm2 inhibitor Nutlin, *J. Biol. Chem* **281**, 33030-33035 (2006).

S4. Tovar, C., Rosinski, J., Filipovic, Z., Higgins, B., Kolinsky, K., Hilton, H., Zhao, X., Vu, B.T., Qing, W., Packman, K., Myklebost, O., Heimbrook, D.C., Vassilev, L.T., Small-molecule MDM2 antagonists reveal aberrant p53 signaling in cancer: Implications for therapy, *Proc. Nat. Acad. Science* **103**, 1888-1893 (2006).

S5. Michaelis, M., Rothweiler, F., Klassert, D., Von Deimling, A., Weber,K., Feshe, B., Kammerer, B., Doerr, H.W., Cinatl, J. Reversal of P-glycoprotein-mediated multidrug resistance by the muring double minute 2 antagonist nutlin-3, *Cancer Res*. **69**, 416-421 (2009).

S6. Hu, B., Gilkes, D.M., Farooqi, B., Sebti, S., Chen, J., MDMX overexpression prevents p53 activation by the Mdm2 inhibitor Nutlin, *J. Biol. Chem*., **281**, 33030-33035 (2006).

S7. Harris, C.C. Protein-protein interactions for cancer therapy, *Proc. Nat. Acad. Science,* **103**, 1888-1893 (2006).

S8. Wade, M., Rodewald, L.W., Espinosa, J.M., Wahl, G.M., BH3 activation blocks Hdmx suppression of apoptosis and cooperates with Nutlin to induce cell death. *Cell Cycle* **7,** 1973 - 1982 (2008).

S9. Liu, W.F., Bodmer,Y., Analysis of p53 MUTAtions and their expression in 56 colorectal cancer cell lines, *Proc Natl Acad Sci U S A* **103**, 976-981 (2006).

S10. Shangary, S., Ding, K., Qiu ,S., Nikoloska-Coleska, Z., Bauer, J., Liu, M. Reactivation of p53 by a specific MDM2 antagonist (MI-43) leads to p21-mediated cell cycle arrest and selective cell death in colon cancer. *Mol. Cancer Ther.* **7**, 1533-1543 (2008).

S11. Supiot, R.P., Hill, R.G., Bristow, R.G., Nutlin-3 radiosensitizes hypoxic prostate cancer cells independent of p53. *Mol. Cancer Ther.* **7**, 993-999 (2008).

S12. Huang, B., Vassilev, L.T., Reduced transcriptional activity in the p53 pathway of senescent cells revealed by the mdm2 antagonist nutlin-3, *Aging*. **1**, 845-854 (2009).

S13. Ambrosini, G., Sambol, E.B., Carvajal, D., Vassilev,L.T., Singer,S., Swartz, G.K. Mouse Double minute antagonist Nutlin-3a enhances chemotherapy induced apoptosis in cancer cells with MUTAnt p53 by activating E2F1, *Oncogene.* **26**, 3473-3481 (2007).

S14. Tokalov,S.V., Abolmaali, N.D. Protection of p53 wild-type cells from taxol by nutlin-3 in the combined lung cancer treatment. *BMC Cancer.* **10**, 1-7 (2010).

S15. Sun, S.H., Zheng, M., Ding, K., Wang, S., Sun, Y. A small molecule that disrupts Mdm2-p53 binding activates p53, induces apoptosis and sensitizes lung cancer cells to chemotherapy, *Cancer Biol. Ther*. **7**, 845-852 (2008).

S16. Cheok, C.F., Dey, A., Lane, D.P., Cyclin-dependent kinase inhibitors sensitize tumor cells to nutlin-induce apoptosis: a potent drug combination, *Mol. Cancer Res*. **5**, 1133-1145 (2007).

S17. Secchiero, P., Grazia de Iasio, M., Gonelli, A., Zauli, G., The MDM2 inhibitor Nutlins as an innovative therapeutic tool for the treatment of haematological malignancies, *Current Pharmaceutical Design* **14,** 2100-2110 (2008).

S18. Lin, S.C., Liu, C.J., Chiu, C.P., Chang, S.M., Lu, S.Y., Chen, Y.J., . Establishment of OC3 oral carcinoma cell line and identification of NF-kappa B activation responses to areca nut extract, *J. Oral Pathol. Med.* **33**, 79-86 (2004).

S19. Kudo, Y., Ogawa, I., Kitagawa, M., Kitajima, S., Samadarani Siriwardena, B.S., Aobara, N., Matsuda, C., Miyauchi, M., Takata, T., Establishment and characterization of a spindle cell squamous carcinoma cell line, *J Oral Pathol Med*. **35**, 479-483 (2006).

S20. Coll-Mulet, L.I., Iglesias-Serret, D., Santidrian, A.F., Cosialls, A.M., Frias, M., Castaño, E., Campas, C., Barragan, M., Fernández de Sevilla, A., Domingo, A., Vassilev, L.T., Pons, G., Gil, J., MDM2 antagonist activate p53 and synergize with genotoxic drugs in B-cell chronic lymphocytic leukemia cells. *Neoplasia.* **107**, 4109-4114 (2006).

S21. Sturm, I ., Bosanquet, A.G., Hermann, S., Guner, D., Dorken, B., Daniel, P.T., MUTAtion of p53 and consequtive selective drug resistance in B-CLL occurs as a consequence of prior DNA-damaging chemotherapy, *Cell Death Differ*. **10**, 477-484 (2003).

S22. Zauli, G., di Iasio, M.G., Seccherio, P., Dal Bo, M., Marconi, D., Bomben R. *et al.*, Exposure of B cell chronic lymphocytic leukemia (B-CLL) cells to Nutlin-3 induces a characteristic gene expression profile, which correlates with Nutlin-3-mediated cytotoxicity, *Curr. Cancer Drug Targets* **9**, 510 (2009).

S23. Laurie, N.A., Donovan , S.L., Zhang, J., Shih, C.S., Fuller, C.E., Teunisse, A., Johnson, D.A., Wilson, M.W., Rodriguez-Galindo,C., Quarto, M., *et al.,* Inactivation of the p53 pathway in retinoblastoma, *Nature* **444**, 61-66 (2006).

S24. Elison, J.R., Cobrinik, D., Claros, N., Abramson, D.H., Lee, T.C. Small molecule inhibition of HDM2 leads to p53-mediated cell death in retinoblastoma cells, *Arch. Ophtalmol.* **124,** 1269-1275 (2006).

S25. Zheng, T., Wang, J., Song, X., Meng, X., Pan,S., Jiang,H., Liu,L. Nutlin-3 cooperates with doxorubicin to induce apoptosis of human hepatocellular carcinoma cells through p53 or p73 signaling pathways. *J. Cancer Res. Clin. Oncol*. **136,** 1597-604 (2010).

S26. Jiang, M., Pabla, N., Murhpy, R.F., Yang, T., Yin, X.M., Degenhardt, K., White, E., Dong, Z. Nultin-3 protects kidney cells during cisplatine therapy by suppressing Bax/Bak activation, *J. Biol. Chem*. **282**, 2635-2645 (2007).

S27. Van Maerken, T., Ferdinande, L., Taildeman, J., Lambertz, I., Yigit, N., Vercruysse, L., A. Rihani,A., Michaelis, M., Jr Cinatl,J., Cuvelier, C.A., Marine, J.C., De Paepe, A., Bracke, M., Speleman, F., Vandesompele, J., Antitumor activity of the selective mdm2 antagonist nutlin-3 against chemoresistant neuroblastoma with wild-type p53. *J. Natl. Cancer Inst*., **101**, 1562-1574 (2009).

S28. Zhu, N., Gu, L., Zhou, M., Inhibition of Akt/surviving pathway synergizes the anti-leukemia effect of nutlin-3 in acute lymphoblastic leukemia cells, *Mol. Cancer Ther*. **7**, 1101-1109 (2008).

S29. Gu, L., Zhu, N., Findley, H.W., Zhou,M. Mdm2 antagonist nutlin-3 is a potent inducer of apoptosis in pediatric acute lymphoblastic leukemia cells with wild-type p53 and over-expression of Mdm2*. Leukemia* **22**, 730-739 (2008).

S30. Drakos, E., Thomaides, A., Medeiros, L.J., Leventaki, V., Konopleva, M., Andreeff, M., Rassidakis, G.Z. Inhibition of p53-muring double minute 2 interaction by nutlin-3A stabilizes p53 and induces cell cycle arrest and apoptosis in Hodgkin lymphoma, *Clin. Cancer Res*. **13**, 3380-3387 (2008).

S31. Tabe, Y., Sebasigari, D., Jin, J., Rudelius, M., Davies-Hil, T., Miyake, K., Milda, T., S. Pittaluga,S., Raffeld, M.. Mdm2 antagonist nutlin-3 displays antiproliferative and proapoptotic activity in mantle cell lymphoma, *Clin. Cancer Res*. **15**, 933-942 (2009).
